# The Contribution of RNA Decay Quantitative Trait Loci to Inter-Individual Variation in Steady-State Gene Expression Levels

**DOI:** 10.1371/journal.pgen.1003000

**Published:** 2012-10-11

**Authors:** Athma A. Pai, Carolyn E. Cain, Orna Mizrahi-Man, Sherryl De Leon, Noah Lewellen, Jean-Baptiste Veyrieras, Jacob F. Degner, Daniel J. Gaffney, Joseph K. Pickrell, Matthew Stephens, Jonathan K. Pritchard, Yoav Gilad

**Affiliations:** 1 Department of Human Genetics, University of Chicago, Chicago, Illinois, United States of America; 2 Howard Hughes Medical Institute, University of Chicago, Chicago, Illinois, United States of America; 3 BioMiningLabs, Lyon, France; 4 Committee on Genetics, Genomics, and Systems Biology, University of Chicago, Chicago, Illinois, United States of America; 5 Department of Statistics, University of Chicago, Chicago, Illinois, United States of America; Georgia Institute of Technology, United States of America

## Abstract

Recent gene expression QTL (eQTL) mapping studies have provided considerable insight into the genetic basis for inter-individual regulatory variation. However, a limitation of all eQTL studies to date, which have used measurements of steady-state gene expression levels, is the inability to directly distinguish between variation in transcription and decay rates. To address this gap, we performed a genome-wide study of variation in gene-specific mRNA decay rates across individuals. Using a time-course study design, we estimated mRNA decay rates for over 16,000 genes in 70 Yoruban HapMap lymphoblastoid cell lines (LCLs), for which extensive genotyping data are available. Considering mRNA decay rates across genes, we found that: (*i*) as expected, highly expressed genes are generally associated with lower mRNA decay rates, (*ii*) genes with rapid mRNA decay rates are enriched with putative binding sites for miRNA and RNA binding proteins, and (*iii*) genes with similar functional roles tend to exhibit correlated rates of mRNA decay. Focusing on variation in mRNA decay across individuals, we estimate that steady-state expression levels are significantly correlated with variation in decay rates in 10% of genes. Somewhat counter-intuitively, for about half of these genes, higher expression is associated with *faster* decay rates, possibly due to a coupling of mRNA decay with transcriptional processes in genes involved in rapid cellular responses. Finally, we used these data to map genetic variation that is specifically associated with variation in mRNA decay rates across individuals. We found 195 such loci, which we named RNA decay quantitative trait loci (“rdQTLs”). All the observed rdQTLs are located near the regulated genes and therefore are assumed to act in *cis*. By analyzing our data within the context of known steady-state eQTLs, we estimate that a substantial fraction of eQTLs are associated with inter-individual variation in mRNA decay rates.

## Author Summary

Recent studies of functional genetic variation in humans have identified numerous loci that are associated with variation in gene expression levels, called expression quantitative trait loci (eQTLs). The mechanisms by which these loci affect gene expression, however, are still largely unknown. Specifically, since most studies rely on measures of steady-state gene expression levels, they are unable to distinguish between the relative influences of either transcriptional- or decay-related processes. To address this gap, we examined the specific impact of mRNA decay processes on steady-state gene expression levels for over 16,000 genes in human lymphoblastoid cell lines. By characterizing decay rates in 70 individuals, we show that steady-state expression levels are significantly influenced by variation in decay rates for 10% of genes. Yet, for roughly half of these genes, we find that individuals with higher expression levels also have faster decay rates. This pattern points to a non-simple mechanistic interplay between transcriptional and decay processes, especially for genes involved in rapid cellular responses. Finally, we identify 195 genetic variants that are significantly associated with both gene expression variation and variation in mRNA decay rates. Using these data, we estimate that that a substantial fraction of eQTLs are associated with inter-individual variation in mRNA decay rates.

## Introduction

Substantial variation in gene expression levels exists in natural populations [1]–[5]. Over the past decade, we have learned that much of this inter-individual regulatory variation is associated with specific genetic polymorphisms, which can be identified by mapping expression quantitative trait loci (eQTLs) [6]–[10]. Expression QTL mapping studies in different organisms have led to important insights into the genetic basis for gene regulation and, in a number of cases, into the mechanistic basis for complex phenotypes. In particular, recent eQTL mapping studies in humans have identified thousands of genetic variants affecting gene expression levels [11]–[14], some of which are loci that are also associated with complex diseases [15]–[18]. Nearly all human eQTLs, regardless of the tissue in which they were found, have been identified near the regulated genes and hence are assumed to act in *cis*. A partial explanation for the relatively small number of *trans* eQTLs that have been identified is the low power to map such loci compared to *cis* acting eQTLs (due to the stringent significance criteria required to avoid false positives when mapping across the entire genome, and generally small effect sizes of *trans*-QTLs [8], [19]–[25]).

Despite the recent success in mapping gene expression phenotypes, we still know little about the specific regulatory mechanisms that underlie eQTLs [26]–[29]. Partly, this gap is being addressed by a growing number of large-scale mapping studies of inter-individual variation in genetic and epigenetic regulatory mechanisms (which complement studies of gene expression variation [13], [30]–[34]). Yet, even by incorporating such studies, the processes underlying regulatory variation and their relative importance remain difficult to infer, because all eQTL studies to date – regardless of the model system or species - have relied on measures of steady-state gene expression levels.

Steady-state gene expression levels are generally the result of two opposing biological processes: mRNA transcription, which includes transcript initiation, elongation, and processing, and mRNA decay, which includes spontaneous and targeted degradation of transcripts, as well as dilution [35], [36]. Using only measurements of steady-state gene expression levels, it is impossible to determine the relative contribution of variation in transcription rates and mRNA decay rates to overall regulatory variation. In other words, without additional data, the particular mechanisms underlying steady-state expression level QTLs cannot be inferred with confidence.

To better understand the basis for variation in steady-state gene expression levels requires data on specific aspects of gene regulatory mechanisms. Most recent studies that have done so (though only rarely in the context of QTL mapping), have focused on understanding transcriptional processes contributing to gene expression variation, such as splicing, DNA methylation, histone modification, chromatin accessibility, and transcription factor binding. Results from this emerging body of work indicate that although transcriptional processes contribute substantially to steady-state measurements of gene expression, neither the independent or combinatorial effects of these mechanisms can completely account for variation in steady-state gene expression levels [28], [29], [37], [38]. It is likely that a better account of regulatory variation can be obtained once transcription initiation and RNA decay mechanisms are considered together.

While the details of transcriptional regulation are becoming increasingly understood, the mechanisms influencing variation in mRNA decay rates have thus far received less attention, particularly in mammalian systems [11], [37]–[39]. This bias may reflect the prevalent assumption that transcription initiation rates are the major determinants of overall gene expression levels [40]–[43]. Yet, a few recent studies of mRNA decay mechanisms have challenged this historical view [3], [33], [44]–[47]. In particular, it has been argued that the regulation of mRNA decay processes might be a key determinant of the expression patterns of a large subset of genes. Recent studies in eukaryotic cells have revealed a wide variability of mRNA decay rates across transcripts – with individual mRNA half-lives ranging from a few minutes to several hours – which can often be tied to differences in the functional role of the regulated genes [44], [48]–[50]. For example, studies in yeast, worms, plants, and human primary cells have all found that genes involved in the regulation of transcription tend to produce mRNA that decays faster than mRNA from genes involved in cell cycle or metabolic pathways [1], [3], [41], [48], [51], [52]. Furthermore, the steady-state mRNA levels of the lowest or highest expressed genes are strongly correlated with mRNA decay rates [41], [44], [49], [50], suggesting that in these cases, regulation of mRNA decay is likely an important determinant of gene expression levels.

A number of mechanisms are known to contribute to variation in mRNA decay rates among genes. These include the roles of certain RNA-binding factors such as small RNAs, RNA-binding proteins, and larger RNA-binding complexes, all of which have been shown to bind to both general (such as the AU-rich 3′ untranslated region elements; AREs [15], [26], [53]) and specific RNA motifs [11], [37], [54]. For example, many RNA-binding small RNAs, including miRNAs, have been shown to expedite decay of specific transcripts by creating double stranded RNA that is targeted for degradation by endonuclease enzymes [11], [38], [47]. Similarly, certain interactions between RNA binding proteins and mRNA have been shown to contribute to either higher (“destabilizing proteins”) or lower decay rates (“stabilizing proteins”), though the mechanisms by which they act are not yet fully understood [11], [54], [55]. More generally, we now appreciate that, much like transcription rates, mRNA decay rates are regulated by a combination of *trans* elements (such as proteins, complexes, or small RNAs) binding to a collection of *cis* binding motifs (typically included within the transcript itself) [6], [56], [57]. However, despite increasing understanding about mechanistic details of mRNA decay processes, we still know little about inter-individual variation in mRNA decay rates, in any species.

## Results

We characterized mRNA decay in 70 Yoruba lymphoblastoid cell lines (LCLs) from the HapMap project [36], [58]. These cell lines have been extensively genotyped and/or sequenced at high-depth [40], [59], [60], making them ideal for genetic mapping studies. To determine decay rates, we measured changes in mRNA abundance levels in each cell line at different times after treatment with the RNA elongation complex inhibitor Actinomycin D (ActD), which arrests transcriptional processes. We measured mRNA abundance before treatment (time point 0) and at four time points after treatment (at 0.5 hours, 1 hour, 2 hours, and 4 hours). To account for the decrease in total RNA caused by the ActD treatment over the timecourse experiment, we increased the number of cells from which we extracted RNA as the experiment progressed (Figure S1). We thus were able to hybridize the same amount of mRNA from each time point to an Illumina HT-12 expression microarray. We processed a total of 350 samples over the five time points and seventy cell lines (see Table S1). Our experimental design allowed us to normalize transcript abundance across all 350 arrays using standard approaches (see Methods for more details).

To estimate mRNA decay rates, we fit an exponential decay model to the normalized expression data to obtain estimated gene-specific decay rates for each cell line. Due to our choice of hybridization study design and normalization procedure, all estimated decay rates are relative to the mean cellular mRNA decay rate in the sample, which itself can be estimated by taking into account the number of cells used to extract RNA across the time points (see Methods for more details). We excluded from all further analyses genes that were not detected as expressed even before the arrest of transcription (time point zero) in at least 80% of individuals (see Methods). Overall, we obtained individual-specific estimates of mRNA decay rates for 16,823 Ensembl genes (see Table S1).

### Characterization of genome-wide decay rates

**Figure 1:**
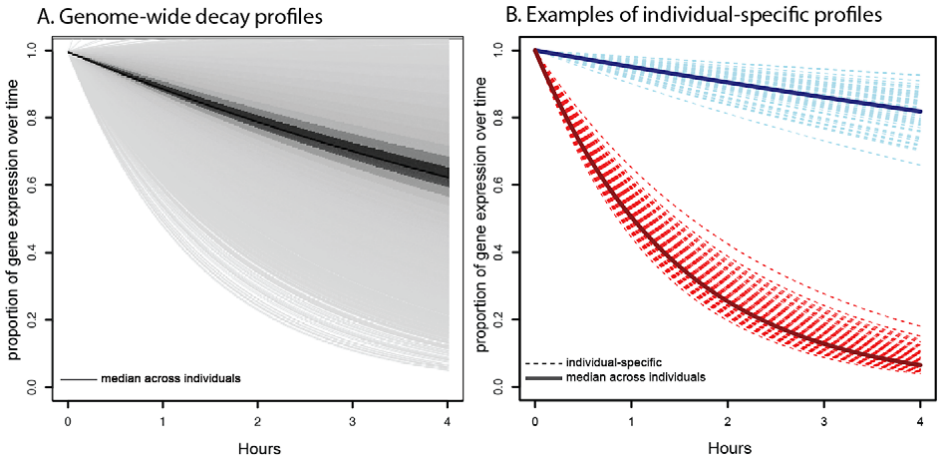
Profiles of decay rates. A. Distribution of genome-wide decay profiles across the timecourse experiment (x-axis), where each decay curve shows the decrease in gene expression level (y-axis) relative to the untreated time point. Each line represents the gene-specific median decay profile, while the darkness of the lines indicates the number of genes sharing that decay profile (darker indicates more genes). B. Representative examples of individual-specific decay profiles (dotted lines) for two genes: *NFKBIE* (in red), which decays faster than average and *DCTN2* (in blue), which decays slower than average. Solid lines indicate the gene-specific median decay profile across all 70 individuals.

As a first step of our analysis, we characterized the genome-wide distribution of mRNA decay rates. To do so, for each gene we used the median decay rate across individuals as a measure of the gene-specific mRNA decay rate. We observed a wide range of mRNA decay rates across genes (Figure 1A), consistent with findings of previous studies. We also observed a substantial amount of variation in decay rates across individuals within each gene (Figure 1B), consistent with expectations from previous studies in human cells [1], [35], [40]. We classified genes as either consistently slow or fast decaying when their decay rates in at least 80% of individuals in our study were classified as slow or fast relative to the individual-mean decay rate (see Methods). We thus identified 146 genes that consistently decayed slower than average across individuals and 716 genes that consistently decayed faster than average.

In agreement with previous observations, we found that genes with related biological functions often decayed at similar rates [1], [52,52]. Genes with slower decay rates tend to be involved in cellular and organelle-related housekeeping processes, such as cytoplasmic and mitochondrial processes (Table S2). Genes with faster decay rates are enriched for gene regulatory functions that might require rapid mRNA decay to ensure rapid turnover of expression levels in response to changing cellular conditions (Table S3). This includes enrichments for functional annotations such as metabolic processes, regulation of gene expression, and regulation of transcription.

**Figure 2:**
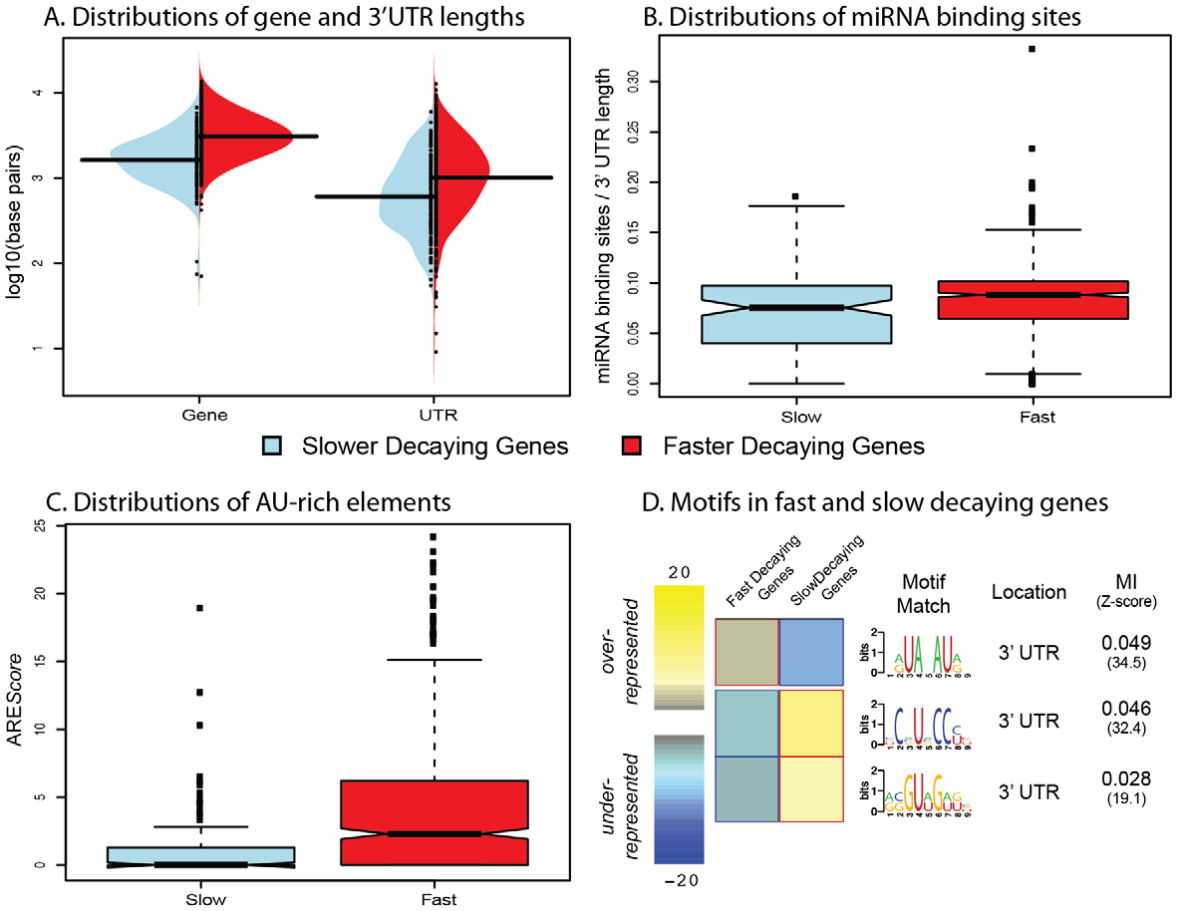
Genomic features influencing variation in decay rates across genes. A. Distributions of gene length (left) and 3′UTR length (right) for genes decaying slower (blue) or faster (red) than average. B. Distributions of the number of miRNA binding sites (normalized by 3′UTR length; y-axis) for genes decaying slower (blue) or faster (red) than average. C. Distributions of ARE*Score*s (y-axis) for genes decaying slower (blue) or faster (red) than average. D. Motifs that are significantly over- (yellow) or under-represented (blue) in fast or slow decaying genes. The MI refers to the mutual information score from the FIRE algorithm.

We next investigated possible mechanisms that could account for variation in mRNA decay rates across genes. Previous studies have suggested that increased transcript length [3], [41], and specifically 3′UTR length [1], [3], might significantly influence mRNA decay rates. Indeed, we find that both are slightly but significantly positively correlated with decay rates across genes (Spearman ρ = 0.17, *P*<10^−16^ for gene length and Spearman ρ = 0.09, *P*<10^−16^ for 3′UTR length). This association is also evident when we limit this analysis only to genes classified as decaying slower or faster than the mean decay rate (Figure 2A; Figure S4; Spearman ρ = 0.15; *P*<10^−16^ for gene length and Spearman ρ = 0.09; *P*<10^−8^ for 3′UTR length). The increased 3′ UTR length in faster decaying genes is thought to indicate an increase in potential regulatory space that could harbor RNA-decay regulatory elements (reviewed in [6]).

Studies of mRNA decay of individual genes have previously identified two main classes of *cis* regulatory elements that might play roles in decay processes: microRNA (miRNA) binding sites [11] and AU-rich elements [15], [17]. To determine the possible influence of miRNA binding on decay rates in the LCLs, we curated several miRNA databases [19], [20], [22]–[25] to create a list of confident miRNA target binding sites (see Methods S1). To account for the confounding effect of transcript length (more binding sites in longer 3′UTRs), we standardized the number of miRNA target binding sites by the 3′UTR length (see Methods). Using this approach, we found a slightly positive correlation between the density of miRNA target sites and decay rates. Again, when we focused exclusively on the genes classified as decaying slower or faster than the mean decay rate, we observed a stronger association (Figure 2B, Spearman ρ = 0.16; *P*<0.003). We then considered the presence of AU-rich elements (AREs) in slower versus faster decaying genes. To do so, we used the ARE*Score* algorithm [26], which searches within 3′UTRs for features associated with typical type-II AREs, to assign an ARE*Score* to each gene. A larger ARE*Score* essentially implies increased potential for binding by an ARE-recognizing RNA binding protein to regulate the decay processes of the gene. We found that there is a significantly increased median ARE*Score* in faster decaying genes compared to slower decaying genes (Figure 2C, Spearman ρ = 0.14; *P*<10^−16^).

As our findings support the general notion that *cis* regulatory elements, such as miRNA bindings sites or AU-rich elements, are important determinants of mRNA decay rates, we next searched for additional sequence motifs that might represent novel binding sites for specific decay factors in LCLs. To do so, we used the FIRE algorithm [30] to search for motifs in the 146 slow decaying genes and 716 fast decaying genes. We identified three significantly enriched motifs – one enriched in the fast decaying genes, and two enriched in the slow decaying genes (Figure 2D). We performed the motif search across the entire promoter and transcript region for each gene, yet all three enriched motifs are located in 3′UTRs. The motif enriched in fast decaying genes closely resembles a typical AU-rich element sequence. The two motifs enriched in slow decaying genes could not be linked to any currently known miRNA seed sequence or RNA-binding protein motif and hence might be novel regulatory elements.

### Relationship between decay rates and steady-state expression levels

**Figure 3:**
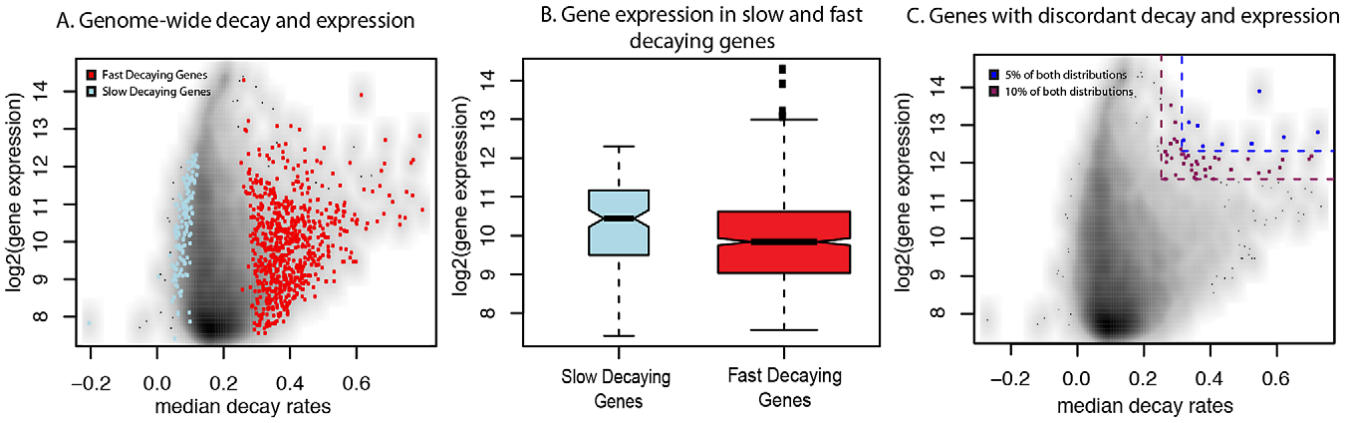
Relationship between gene expression levels and mRNA decay rates across genes. A. Genome-wide scatterplot of median decay rates (x-axis) versus median steady-state expression levels (y-axis) for all genes (black dots, where higher densities are in dark colors), slow decaying genes (blue dots), and fast decaying genes (red dots). B. Genes that are within the top 5% (yellow) or top 10% (blue) of both the decay rate and steady-state gene expression. C. Boxplots of the distribution of steady-state expression levels (y-axis) in genes decaying slower (blue) or faster (red) than average.

We are specifically interested in the effect that mRNA decay has on steady-state expression levels (in these analyses, we defined “steady-state expression” as the mean expression across all time points so that our estimates of steady-state expression levels would be statistically independent of the estimated decay rates when the null hypothesis of no association between steady-state levels and decay rates is true; see Methods). Considering this relationship across all genes (Figure 3A), we found little or no correlation between decay rates and gene expression levels. However, we observed a significant difference in expression levels between genes classified as decaying significantly slower or faster than the mean decay rate (as defined above; *P*<6×10^−6^, Figure 3B; Figure S5). This difference in expression levels is in the expected direction – that is, genes with slower decay rates have higher steady-state expression levels than genes with faster decay rates.

We also observed a small number of cases in which genes with faster decay rates are highly expressed (we refer to this as a ‘discordant’ relationship between gene expression levels and decay rates). One example is the *BTG1* gene, which is involved in regulating the glucocorticoid receptor autoregulatory pathway [35], and has both a significantly increased decay rate and a high expression level (Figure S5). Interestingly, seven of the top nine genes with discordant patterns (both the expression levels and decay rates of these nine genes are within the top 5% of the genome-wide distributions of gene expression and decay rates respectively; Figure 3C; see Methods) have been experimentally shown to be involved in auto-regulatory or regulatory feedback pathways (Table 1) [61]–[69]. More broadly, the top 49 genes with discordant patterns (constituting the top 10% of both the genome-wide distributions of gene expression levels and decay rates; Figure 3C) are enriched for genes with functions related to signaling pathways, stress response, and immune function (when genes expressed in LCLs are used as the background for the analysis; Table S4).

**Table 1:** Genes with discordant decay rates and steady-state gene expression levels.

Gene Name(s)	Ensembl ID	Function	Evidence for Negative Feedback Function (ref[s].)
Dec1, Stra13, BHLHE40	ENSG00000134107	control of cell differentiation and signaling pathways	Autoregulation of gene expression ([53], [54])
BTG1	ENSG00000133639	Regulates cell growth and differentiation	Involved in GR autoregulatory pathway ([44])
CCR7	ENSG00000126353	Mediator of EBV effects on B lymphocytes; activates B and T lymphocytes	*None*
DDIT4	ENSG00000168209	Inhibits cell growth	Negative feedback control of mTOR signaling pathway ([55])
HCP5	ENSG00000206337	Regulates cellular response to stress	*None*
PPP1R15A	ENSG00000087074	Regulates cellular response to stress	Negative feedback loop promoting basal cellular activity ([56])
XBP1	ENSG00000100219	ER stress response element	Autoregulates gene expression ([57], [58])
ZFAND5, ZNF216	ENSG00000107372	Involved in regulation of TNF-induced NF-κB activation	Overexpression leads to apoptosis ([59])
ZFP26	ENSG00000128016	Regulates response to growth factors	Autoregulates mRNA stability ([60], [61])

A list of the 9 genes that are in the top 5% of both the decay rate and steady-state gene expression distributions, thus showing evidence of both fast decay and high expression. The table lists the gene name (column 1), the Ensembl ID (column 2), the function of the gene (column 3), and evidence from the literature pointing towards negative feedback or autoregulatory functions for the gene (column 4).

**Figure 4:**
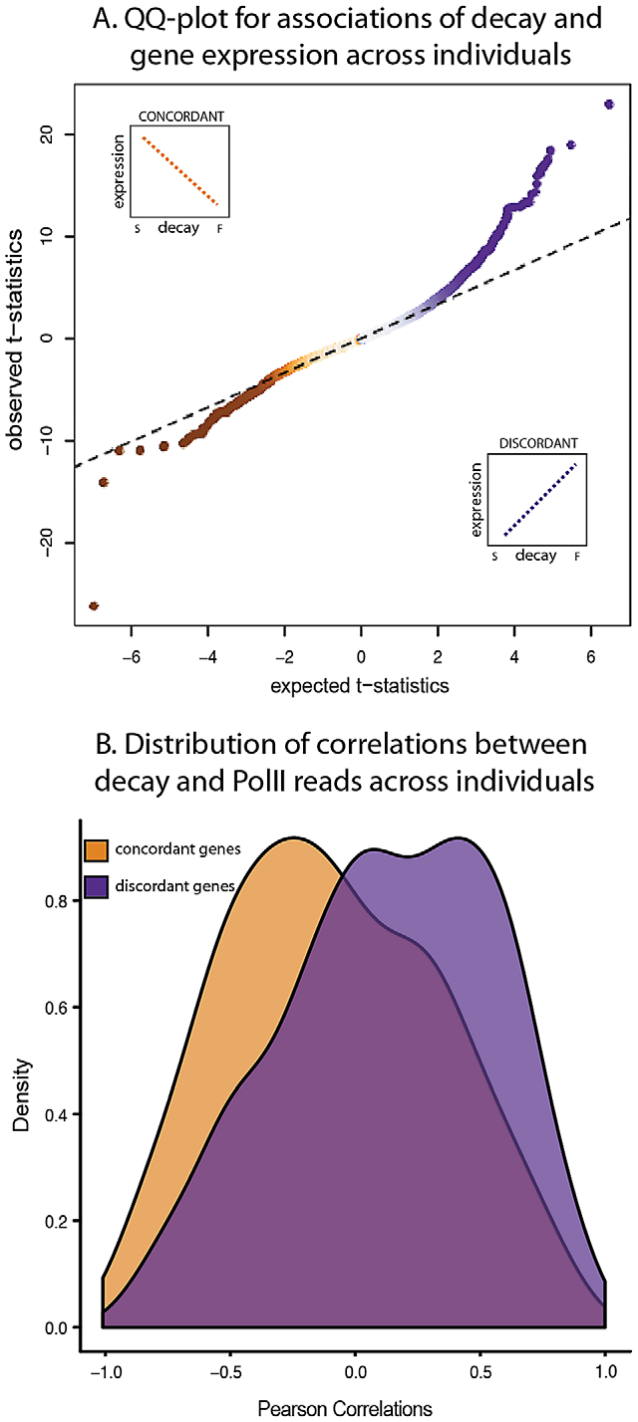
Relationship between gene expression levels and mRNA decay rates across individuals. A. QQ-plot of the t-statistics for association between steady-state expression levels and decay rates across individuals (y-axis) compared to the null distribution of t-statistics assessed by permutations (x-axis). The sign of the t-statistic indicates the direction of correlation. Genes with concordant relationships (orange) have negative t-statistics and genes with discordant relationships (purple) have positive t-statistics. B. Density distributions of the Pearson correlations (x-axis) between mRNA decay rates and PolII reads for genes with either concordant (orange) or discordant (purple) relationships between decay rates and steady-state expression levels across individuals.

We next examined the extent to which variation in decay rates might contribute to overall variation in steady-state expression levels across individuals. For each gene, we calculated the correlation between gene expression levels and mRNA decay rates across individuals and focused on genes with a significant (FDR = 10%) correlation between the two measurements (Figure 4A). We found a significant negative correlation between expression levels and decay rates for 695 genes. It is reasonable to assume that inter-individual variation in steady-state expression levels of these 695 genes is driven by corresponding variation in decay rates. Based on gene ontology functional annotations, these 695 genes are enriched for genes involved in endopeptidase inhibitor and regulator activity (Table S5).

On the other hand, we also found a discordant relationship between gene expression levels and decay rates across individuals for 989 genes (10% FDR; Figure 4A). This finding may seem counter-intuitive as it contradicts our expectation that higher decay rates should result in lower steady-state gene expression levels. However, genes with a discordant relationship between expression and decay are enriched for processes involved in the regulation of cellular, metabolic, and transcriptional activities (Table S6). A similar observation of discordant relationships between decay rates and expression levels that are enriched for genes in the same functional categories (metabolic, and transcriptional activities) has been previously reported in yeast [37], [38]. Put together, these results suggest a role for mRNA decay in complex regulatory circuits that have the property of fast response time, for instance auto-regulation by negative feedback loops.

Studies across yeast species [37], [38] have further suggested that positive correlations between gene expression levels and decay rates are often coupled with correspondingly increased transcription rates – presumably to increase response speed [40]. To test this notion in our system, we used a combination of previously published [33] and newly generated PolII occupancy ChIP-seq data from seven of the same Yoruba LCLs as a proxy measurement of gene-specific transcription rate (Table S7). Our hypothesis, based on the observations from the yeast studies, was that transcription and decay rates are often positively correlated in genes with discordant relationship between expression levels and RNA decay rates across individuals. Indeed, we found a significant increase in positive correlations between transcription and mRNA decay rates for genes with discordant compared to genes with a concordant relationship between expression and decay (*P*<10^−3^; Figure 4B; Figure S6) and compared to the distribution of correlations between transcription and mRNA decay rates of all genes in the data set (*P*<10^−16^).

### Mapping mRNA decay QTLs

**Figure 5:**
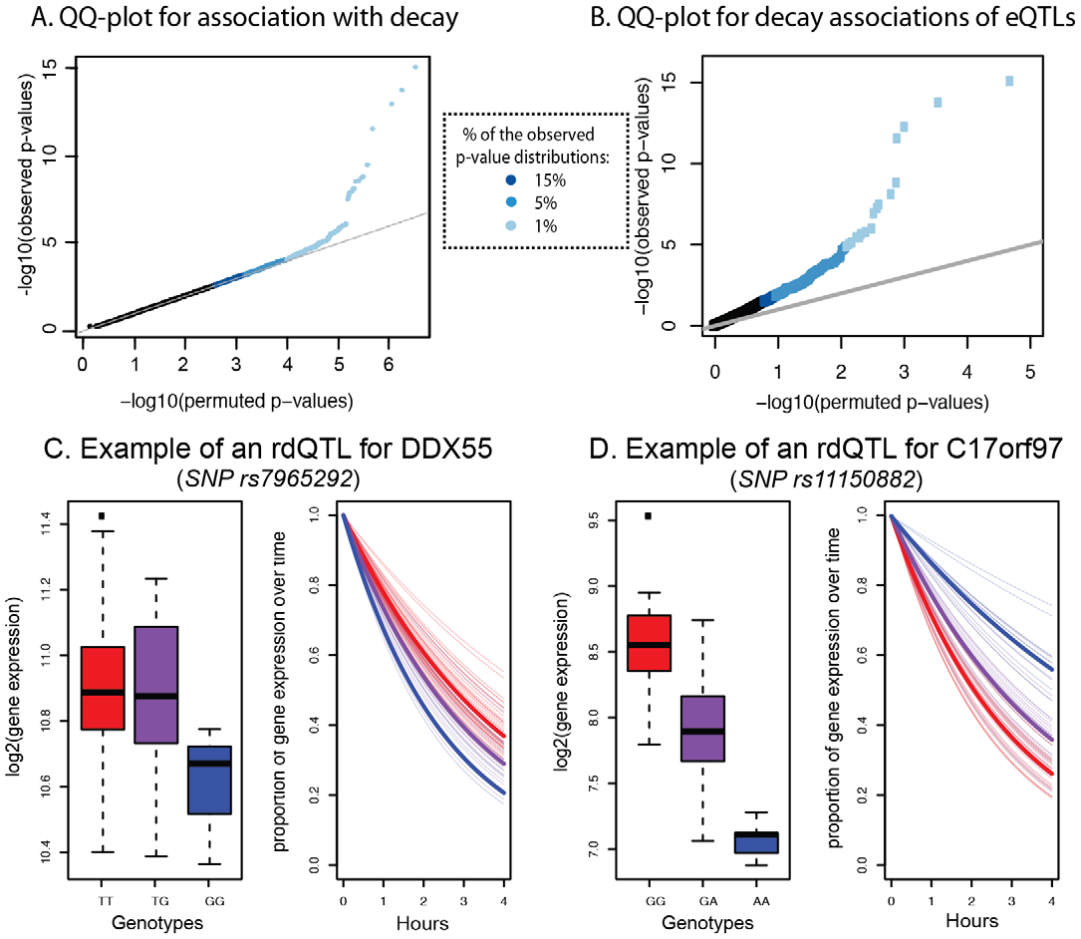
Genome-wide identification of rdQTLs and representative examples. A. QQ-plot for all tests of association between mRNA decay rates and variants within a *cis* region of 25 kb around the target gene (y-axis) compared to a null distribution of p-values based on permutations (x-axis). B. QQ-plot for all tests of association between the most significant eQTL SNP for a gene and the mRNA decay rate for the same gene (y-axis) compared to a null distribution of p-values based on permutations (x-axis). C. Example of an rdQTL with concordant eQTL-rdQTL effects (for the gene *DDX55*). D. Example of an rdQTL with discordant e-QTL-rdQTL effects (for the gene *C17orf97*).

Finally, we investigated the genetic basis for inter-individual variation in mRNA decay rates. To do so, we treated the mRNA decay rates as a quantitative trait and mapped genetic loci influencing variation in this trait. We tested for association between individual-specific estimates of mRNA decay rates and genotypes in a *cis* candidate region of 25 kb centered around the target transcript boundaries. Using this procedure, we found 31 genes with significant RNA decay quantitative trait loci (rdQTLs) at a 15% FDR (Figure 5A). Expanding our mapping procedure to include genome-wide polymorphisms, we found no evidence for significant trans-acting rdQTLs, likely because our experiment is underpowered to detect *trans* effects (see Methods S1).

Given the observed significant correlation between steady-state gene expression levels and decay rates across individuals, we hypothesized that we might have better power to detect more rdQTLs at a given FDR if we focused on SNPs already identified as steady-state expression QTLs. To do so, we first mapped eQTLs using the mean expression data across time points. We identified 1,257 eQTLs (at 15% FDR; see Methods), most of which were previously observed in these cell lines. Within this set, 195 (16%) of the eQTLs were also significantly (at 15% FDR) associated with variation in mRNA decay rates (Figure 5B, Table S8). In other words, 195 of the steady-state gene expression QTLs are also classified as rdQTLs using our approach; a significant enrichment of decay effects compared to that expected by chance (*P*<0.001). Using the method of Storey *et al.* to conservatively estimate the proportion of tests where the null hypothesis is false (while accounting for incomplete power [48]), we estimate that 35% of the most significant eQTL SNPs are also associated with decay rates (Figure S7).

**Figure 6:**
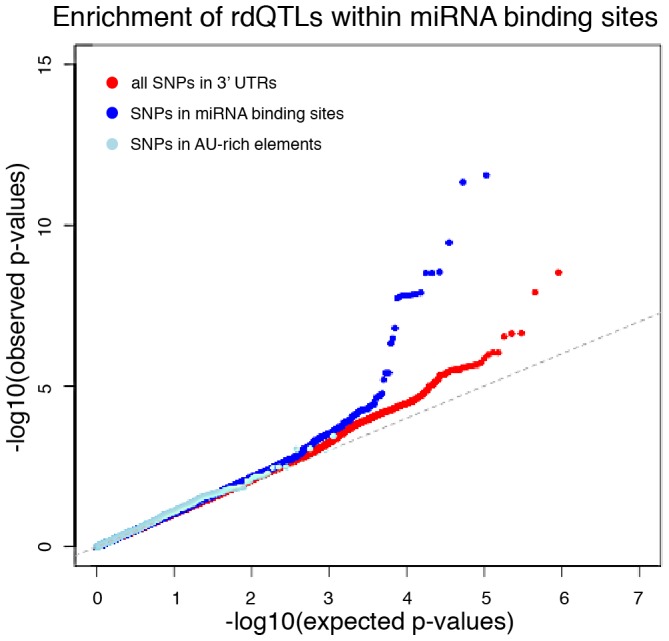
miRNA binding sites are enriched with rdQTLs. The QQ-plots of expected versus observed quantiles of the –log10(p-values) testing the null hypothesis that there is no association between the SNP and RNA decay, for all 3′UTR SNPs (red) and in two known 3′UTR functional annotations – predicted miRNA binding sites (dark blue) and AU-rich element pentamers (light blue).

We asked whether SNPs that are identified as rdQTLs are enriched in particular genomic annotations, especially when compared to eQTL SNPs. Since our mapping approach does not allow us to identify with confidence the causal site, we proceeded by considering and comparing the strength of association with decay rates across SNPs in different genomic annotations. Using this approach we found that, in general, the same functional annotations that were previously found to be enriched for eQTLs are also enriched for rdQTLs (e.g., exons, UTRs, and promoter regions; Figure S8A). Yet, while eQTL are generally enriched in 3′ UTRs (Figure S8B), rdQTLs are specifically enriched in predicted miRNA binding sites within 3′ UTRs (Figure 6). This observation is consistent with the hypothesized importance of miRNA-mediated regulation of mRNA decay.

### Explaining variation in gene expression levels

We next examined the relationship between eQTLs and rdQTLs in more detail. We found that in the majority of the joint QTLs (55%), the allele that is associated with lower steady-state expression level is also associated with faster mRNA decay rate, as expected if differences in decay rates drive differences in expression levels across individuals (Figure 5C). However, in the remaining 45% of cases, the allele that is associated with lower gene expression levels is associated with slower mRNA decay rates (Figure 5D). This implies a more complicated regulatory mechanism, which counters the effect of decay at these loci to drive opposite patterns of gene expression across individuals (see Discussion). We thus focused only on the 55% of eQTLs-rdQTL sites with concordant genotypic effects, for which a more intuitive and simple mechanistic explanation is likely. We again used the method of Storey *et al.* [48] and estimated that as many as 19% (95% CI by bootstrapping: 15%–21%) of eQTLs might be regulated, at least in part, by differences in decay rates. We acknowledge that (as with any comparison and combination of results from genome-wide mapping studies) any factor that affects the power to find associations may result in a biased estimate of the proportion of eQTLs that are also classified as rdQTLs. It is unclear how one could identify and test for all possible relevant factors. In our analysis, we have taken into account the possible effect of overall gene expression levels on eQTL/rdQTL mapping (see Methods), and confirmed that the distributions of the number of SNPs in the proximal window are similar whether one considers sites classified as either eQTLs only or as eQTLs/rdQTLs (Figure S9). On the other hand, we did find a difference in the distribution of minor allele frequency, and the distributions of the number of individuals that are homozygote to the minor allele, between eQTLs and eQTLs/rdQTLs (Figure S9), but this would be conservative with respect to the estimated proportion of eQTLs that are also rdQTLs (namely, the true overlap might be higher than 19%).

Using a similar approach, we have previously found that up to 55% of eQTLs might be explained by variation in DNase sensitivity (these eQTLs were also classified as dsQTLs [32]). We expected that the combination of RNA decay data and DNase sensitivity profiles might explain a larger proportion of inter-individual variation in gene expression levels. To test this using LCLs from the 66 individuals used in both the DNase sensitivity [32] and the current study, we first examined the overlap between SNPs identified as either eQTLs, rdQTLs or dsQTLs. In order to standardize the analyses, we re-mapped eQTLs, rdQTLs, and dsQTLs using only the set of 66 YRI LCLs used in both our study and Degner *et al.* [32]. We identified 1,147 eQTLs (15% FDR), of which 171 were also classified as rdQTLs (15% FDR) and 168 as dsQTLs (15% FDR; Figure S10). There is a slight enrichment in the overlap of eQTLs classified as both rdQTLs and dsQTLs (33 SNPs; 25 are expected by chance along; *P* = 0.03). This might reflect variation that affects gene expression levels through coupled transcription and decay processes.

Put together, 26.7% eQTLs are also classified as either rdQTLs and/or dsQTLs. Combining all three annotations (see Methods; Figure S11) we estimated (by using the Storey method [48])that up to 63% of eQTLs could be driven, at least in part, by either decay or chromatin accessibility-related mechanisms. We note that for this comparison we are including both concordant and discordant rdQTLs, since both patterns could be representative of either simpler or complex mechanisms underlying gene expression variation.

## Discussion

We conducted a genome-wide study of inter-individual variation in mRNA decay levels in 70 human LCLs to investigate the extent to which variation in mRNA decay might account for overall gene expression variation. Our observations, both across genes as well as across individuals, lend support to the notion that regulation by decay processes is a significant mechanism by which steady-state transcript levels are modulated.

Consistent with previous studies, we found substantial variation in mRNA decay rates across genes [44], [49], [50]. We caution that the experiments to obtain decay rates involve treatment with an antibiotic (ActD), which is toxic to cells and may therefore be associated with certain artifacts. That said, ActD is a well-established reagent for studies of this type and the conditions we used here closely reflect those of earlier studies of mammalian mRNA decay. One inherent limitation of our study design is the inability to calculate absolute decay rates and thus mRNA half-lives. Instead, we were only able to estimate decay rates relative to the mean cellular mRNA decay rate. Using data collected using commercial microarrays (rather than, for example, RNA sequencing data), this was the only way we were able to normalize the data across time points without making explicit assumptions regarding the distribution of decay rates. Our normalization approach allowed us to maintain the relative order of genome-wide decay rates across genes and individuals. Yet, it also likely resulted in a limited range of the estimated variance of decay rates across genes compared to the true underlying distribution of absolute decay rates. Thus, the results and analyses presented here may underestimate the magnitude of variation in mRNA decay rates across genes.

### The relationship between RNA decay and steady-state gene expression levels

In many cases, our observations across genes were consistent with the intuitive model whereby faster mRNA decay rates are associated with lower steady-state gene expression levels. Accordingly, we observed lower and higher steady-state gene expression levels for the most rapidly and slowly decaying genes, respectively. Focusing only on these intuitively simple regulatory interactions across QTLs, we estimated that up to 19% of eQTLs might influence gene expression variation through an effect on mRNA decay rates. Incorporating rdQTLs with data on DNase sensitivity QTLs (dsQTLs), we estimated that a combination of variation in RNA decay rates and chromatin accessibility might explain the majority (63%) of eQTL effects. In addition, we find that SNPs within miRNA binding sites show an enrichment for association with variation in decay rates compared to all 3′UTR SNPs, leading to a hypothesis that variation in miRNA binding plays a particularly important role in regulating decay rate variation.

Interestingly, however, we observed many instances of the opposite (discordant) relationship between mRNA decay rates and steady-state gene expression levels. Overall, 59% of genes with a significant correlation between decay rates and expression levels across individuals show a discordant relationship (though only 45% of eQTL/rdQTL pairs). The frequency of this phenomenon seems somewhat unexpected especially given the stronger overall concordant relationship between decay and expression when all genes are considered. It may also cast doubt on the mechanistic explanation we offered for the more intuitive – concordant – relationship between RNA decay and gene expression levels. On the other hand, prevalent discordant decay rates and expression levels across genes have been previously observed in yeast. We speculate that these discordant patterns are the result of complex regulatory circuits, which have evolved to address the need for shorter response time or to stabilize steady-state gene expression levels within the cell. Indeed, the majority of genes with discordant decay and expression patterns are known to be involved in biological processes that require fast response time (Table S3). In a subset of these cases, an auto-regulatory or regulatory feedback circuit has been demonstrated (Table 1). Since many stress and immune response pathways are activated (namely, these genes are highly expressed [53]) in LCLs due to the EBV infection which causes immortalization, we hypothesize that we were able to identify discordant patterns of decay and gene expression at a higher frequency than otherwise expected in resting cells.

Discordant differences in the rates of transcription and mRNA decay could be achieved by a coupling of decay and transcriptional regulatory mechanisms. Dori-Bachash and colleagues suggested that discordant patterns between two closely related yeast species might be due to such coupling whereby the same *cis* elements may regulate both processes [37]. Supporting these findings, Shalem *et al.* found that PolII binding in yeast could regulate coordinated mRNA synthesis and degradation processes [38], building on work from Harel-Sharvit *et al.* that implicated PolII as a factor linking both transcription and mRNA decay to translation in yeast [55]. Additional evidence has pointed to an intrinsic role for the same promoter binding elements promoting both mRNA synthesis in the nucleus and mRNA degradation in the cytoplasm [56], [57]. Our observations also lend support to an explanation based on coupling of the transcription and RNA decay processes.

Such mechanistic coordination implies complex regulatory circuitry, which suggests that decay processes might be playing an important role in maintaining an upper limit of steady-state gene expression, while allowing for rapid transcriptional response - a classical auto-regulatory feedback loop motif [36]. Coupling different regulatory mechanisms to cause such regulatory motifs has been suggested as a way by which cells optimize systems-level functionalities [40]. This is especially important in the context of transcriptional responses to external stimuli or stress. In these situations, coupling of transcription and mRNA decay might be an efficient strategy that allows rapid and precise control of cellular response to external perturbations [40].

Previous studies provided evidence for the important role of mRNA decay in regulating cellular response. For instance, Raghavan *et al.* found that activation-induced genes in human T-lymphocytes cells, which are enriched for transcriptional regulatory functions, tend to have fast decay rates [52]. Shalem and colleagues evaluated changes in mRNA decay and transcription rates in yeast subjected to either transient or enduring stresses [70]. Yeast subjected to the enduring stress displayed an expected behavior whereby most induced genes were stabilized, while under the transient stress, most induced genes exhibited faster decay rates regardless of their increased steady-state expression levels [70]. Our rdQTL data suggest that variation in regulatory elements that affect mRNA decay rates may play an important role in the individual-specific efficiency of response regulatory circuitry.

### Summary

We have taken some of the first steps towards characterizing the impact of variation in mRNA decay rates on variation in gene expression levels. Our results indicate that decay processes might play a crucial role in fine-tuned genome-wide regulation of gene expression variation in humans. In particular, we found that a moderate proportion of eQTLs might be due to variation in decay rates, and that negative feedback regulatory circuits involving mRNA decay processes may be common in humans. Further study of the mechanisms underlying variation in mRNA decay rates is needed to increase our understanding of the genetic basis of steady-state gene expression levels and the underlying regulatory circuits.

## Methods

### Cell culture, Actinomycin D treatment, RNA isolation

Cell lines were grown using standard procedures (as recommended by Coriell) by culturing cells in RPMI 1640 (supplemented with 2 mM L-glutamine and 15% fetal bovine serum). Each of the cell lines was treated with Actinomycin D (ActD) to inhibit transcription, with one biological replicate from each cell line. Because ActD terminates active transcript elongation by binding directly to DNA in a reversible manner [12]–[14], [71], [72], it is generally thought to be the most effective transcriptional inhibitor [16], [18], [72]–[74]. ActD treatment was performed by culturing cells at a concentration of 750,000 cell/ml with 7.5 ug/ml of ActD.

Based on the results from a pilot experiment (see Methods S1, Figure S1, Figure S2, Figure S3), we extracted RNA at a total of five timepoints: before the treatment with ActD (0 hours) and after treatment (0.5 hours, 1 hour, 2 hours, and 4 hours). To account for the decrease in total RNA resulting from the treatment and to obtain enough RNA from each timepoint for subsequent microarray hybridization, we increased the number of cells from which we extracted RNA over the timecourse (Figure S1). Total RNA was extracted using an RNeasy Mini Kit (Qiagen) and RNA quality was assessed using an Agilent Bioanalyzer.

### Microarray analysis and normalization

We estimated gene expression levels in all samples (350 total samples across all 5 time points and 70 cell lines) by hybridizing RNA to the Illumina HT-12 v4. Expression BeadChip arrays. As RNA yield is expected to change across samples from different time points (due to RNA decay), previous microarray based studies of RNA decay have typically normalized their data using spiked-in samples [3], [8], [21]. The Illumina HT-12 arrays, however, do not include non-human probes that would allow us to use spike-ins. Instead, we hybridized the same quantity of RNA from each time point to the microarrays using standard Illumina hybridization protocols. Subsequently, we normalized the array data using standard approaches across all the arrays [27]–[29], [75], [76].

All low-level microarray analyses were performed in R using the Bioconductor software package *lumi* [13], [31]–[34], [77]. Specifically, we performed a log2 variance stabilizing transformation and robust spline normalization (RSN). Following normalization, we removed probes with intensities indistinguishable from background noise in either the 0 and/or 4 hour time points on the array (as measured by the negative controls present on each array). In addition, we mapped the Illumina 50 bp probe sequences using BWA v.0.4.6 [36], [78] and retained only probes that mapped uniquely with 100% identity to an exon within an annotated gene from the Ensembl database (2009-12-31 version). Following filtering based on detection and probe mapping (see Supplemental Materials), data from 23,065 probes corresponding to 16,823 genes were used for all further analyses. For gene-based analyses, we considered the mean expression across the set of probes corresponding to a single gene as the expression level of that gene. For all genotype analyses, SNPs located within probes could bias probe hybridization and downstream measures of steady-state gene expression across individuals. For the 3,327 probes overlapping one or more SNPs, we aimed to remove the effect of SNPs on probe hybridization by regressing steady-state expression levels on the genotype of the SNP located within the probe. In cases where this regression was significant (*P*<0.05), we used the residual of the regression as the steady-state expression measurement [28], [29], [79].

After all normalization and filtering steps, genes whose transcripts decayed at an “average” rate appeared to be expressed at a constant level through the timecourse measurements (Figure S2). For ease of visualization, the expression levels across time points in all decay profiles plotted throughout this manuscript have been standardized by the total number of cells from which RNA was extracted (Figure S1).

### Calculation of mRNA decay rates (and fast/slow decaying genes)

Because mRNA decay has been shown to exhibit properties of first-order decay [11], [39], [80], [81], we estimated gene-specific RNA decay rates in each cell line by using a regression equation of the form (a linear transform of the first-order exponential decay model):
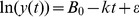
(1)where *y(t)* is the mRNA abundance at time *t*, *B_0_* is the mRNA abundance at the untreated time point (time point ‘0’), *k* is a gene-specific decay rate constant, and variance ε∼N(0,σ^2^). For subsequent analyses, we used the gene-specific decay rate constant *k* as an estimate of a decay rate. Under these conditions, genes with decay rates close or equivalent to the mean cellular decay rate are represented by *k* = 0. To identify genes that decay significantly faster or significantly slower than the mean mRNA decay rate in LCLs, we identified genes for which *k* significantly differed from zero (mean decay rate). We fit gene-wise decay rates for each cell line and identified genes for which least 80% of individuals had estimated values of k that differed significantly from 0 (*P*<0.1) in the same direction (either faster or slower decay than the mean decay rate).

To rank genes by their combined gene expression and decay values, we examined the genome-wide distributions. For example, genes with discordant patterns are those with high (or low) expression levels and whose mRNA decays rapidly (or slowly). To classify such patterns, we independently identified genes within the top 5% and 10% tails of the decay rate and steady-state gene expression distributions and then considered the overlaps across the two data sets (Figure S5B). We identified 9 and 49 genes at the top 5% and 10%, respectively, of both the gene expression and decay rate distributions.

### Determination of genomic annotations

To determine the effect of gene length and 3′UTR length on mRNA decay rates, gene lengths and 3′UTR lengths were calculated using information extracted from the Ensembl gene database (2009-12-31 version). [41]–[43], [82]. Total gene length was defined as the distance between the upstream most TSS and the downstream most transcription end site (inclusive of both exons and introns). Total 3′UTR length was calculated as the number of bases annotated as being within a 3′UTR in any isoform of the given gene.

In order to create a comprehensive set of microRNA (miRNA) binding site predictions, we downloaded the miRNA binding predictions from three databases: microRNA.org, PicTar, and targetScan [3], [19], [20], [22]–[25], [44]–[47]. By parsing the predictions for all miRNAs in these three databases, we obtained a combined set of miRNA predictions that were present in one, two, or all three databases. Because each of these databases uses different sets of annotations and identifiers, we applied a series of conversion and filtering steps for each database (see Methods S1 for details). We used the ARE*Score* algorithm (http://arescore.dkfz.de/arescore.pl) [26], [44], [49], [50] to calculate an ARE*Score* as a proxy for the number of AU-rich elements present in 3′UTRs. The program was run with default parameters on RefSeq defined 3′UTR regions for all genes in our dataset [1], [3], [41], [51], [52], [83].

To identify significantly over- or under-represented motifs in either fast or slow decaying genes, we used the FIRE algorithm (https://tavazoielab.c2b2.columbia.edu/FIRE/) [30], [41]. We tested for motif enrichment in promoter regions and full gene bodies of both fast and slow decaying genes, using default FIRE parameters. In all tests, we compared against a background set of all genes that were present in our study.

### Gene Ontology analyses

We used GeneTrail (http://genetrail.bioinfo.uni-sb.de) [15], [26], [84] to test for enrichments of functional annotations among different classes of genes: (a) genes consistently decaying faster or slower than the mean cellular decay rate, (b) genes at the top 10% of both the gene expression and decay rate genome-wide distributions, and (c) genes showing either concordant or discordant relationships between decay rates and gene expression levels. In all tests, we used a background set of all genes that were present in our study and detected as expressed in either the zero or four hour timepoints. The tests were performed using all GO categories and KEGG pathways. We calculated p-values using a hyper-geometric distribution and report false discovery rates for each p-value.

### Inter-individual correlation between decay rates and expression levels

To investigate the contribution of variation in decay rates to overall variation in steady-state gene expression levels across individuals, we identified genes whose expression levels and decay rates were significantly correlated. Specifically, for each gene, if *y*_i_ denotes the steady-state expression level (defined here as the mean of the expression levels across all time points in order to increase statistical independence from the estimated decay rates) for individual *i* and *r*_i_ denotes the corresponding decay rate estimate, we fit a linear model of the form:
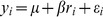
(2)where the coefficient, *β*, measures the strength of the association between decay rate and steady-state gene expression levels. In order to identify genes where the coefficient, *β*, represents a significant association, we repeated the analyses with 3 sets of permuted decay rates, recorded the significance of *β* from each permutation, and used these permuted p-values as an empirical null distribution. We estimated the FDR by comparing the true distribution of p-values of *β* to this null distribution.

### Analysis of PolII ChIP–seq data

PolII ChIP-seq data on six YRI LCLs (GM18505, GM18522, GM19141, GM19193, GM19204, and GM19238) were collected within the context of another study within the lab. ChIP-seq libraries were prepared as described previously [11], [54], [85], using the non PolII antibody H-224 (Santa Cruz Biotechnology, sc-9001x). In addition, raw PolII ChIP-seq reads from a seventh YRI LCL, GM19099, was obtained from a previously published study [11], [33], [47] and analyzed in a similar fashion to the PolII ChIP-seq data generated in-house.

Raw PolII ChIP-seq reads were mapped back to human genome (hg18) using BWA v.0.4.6 [11], [54], [78] and reads from multiple lanes from the same individual were combined into a single mapped file. For each individual, we used Samtools [6], [86] to isolate reads in genic regions (as defined in the Genomic Annotations section above) and promoter regions (defined as 1 kb upstream and 1 kb downstream of the transcription start site). For genic regions, read counts were normalized by the total length of the genic region to be able to compare across genes with varying length. For individual-specific measures of PolII occupancy for each gene, read counts were normalized by the total number of mapped reads per individual.

### Quantitative trait loci (QTL) association mapping

For all QTL mapping analyses, we used close to full genotype information for each of the 70 YRI individuals, achieved by combining available datasets and imputing missing genotypes with the BimBam software [58], [87], [88] as described previously [32], [59], [60]. Briefly, we built a reference panel consisting of the largest set of all 210 YRI HapMap individuals and gathered genotypes for any SNP or short insertion/deletion (indel) called in either HapMap (Release 28; October 2010, [1], [35], [59]) or 1000 Genomes (May 2011 interim phase 1 release, [1], [52], [60]) datasets. Missing genotypes in the individuals in this study were imputed using this reference panel, resulting in a total of approximately 15.8 million variants genome-wide.

All associations between genotypes and either decay rates or gene expression were examined using a linear regression model in which each phenotype was regressed against genotype. For all analyses, we only tested association under the assumption that SNPs affected the resulting phenotype in an additive manner (i.e. heterozygote phenotypic mean equals the average of the two homozygote means). For each gene, we tested for association of the phenotype with the genotypes of SNPs and indels within a *cis*-candidate region of 25 kb around the gene (25 kb upstream of the TSS and 25 kb downstream of the TES). We chose this definition of a *cis*-candidate region to map variation in mRNA decay rates in an unbiased manner by including SNPs outside of transcript regions. Indeed, recent reports have indicated that elements in intergenic promoter elements [56] and RNA binding proteins binding intronic regions [89] could regulate mRNA decay mechanisms. To evaluate genotypic effects on decay variation for a given gene, we tested associations with SNPs or indels with a minimum allele frequency greater than 10%, using the following model:
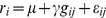
(3)where *r*_i_ is defined as in model (2) and g_ij_ corresponds to the genotype of individual *i* at variant *j*, coded as 0, 1, or 2 copies of the minor allele. In this model, the coefficient *γ* indicates the strength of association between the mRNA decay rate of the gene and genotypes at variant *j*. To estimate the false discovery rate, we permuted phenotypes three times, re-performed the linear regressions, and recorded the minimum p-value (across SNPs/indels) for each gene for each permutation. These sets of minimum p-values were used as our empirical null distribution. We estimated the FDR by comparing the true distribution of the minimum p-values to this null distribution, as previously described. Previous studies mapping cis-associations have found that statistical power to detect associations can be dramatically increased by accounting for unmeasured confounders within quantitative measure of the phenotype [3], [12], [13], [31], [32], [41], [90], [91]. When considering decay as the phenotype, we did so by performing principal components analysis (PCA) on the (70 by 70) correlation matrix of decay rate estimates. We found the strongest rdQTL signal (largest number of findings at a fixed FDR) when 13 principal components (PCs) were regressed out.

When considering steady-state gene expression as the phenotype, we performed all analyses on mean expression levels across all time points per individual in order to reduce the variance of expression measurements and increase the statistical independence between the eQTL estimates and the estimates of decay rates. We quantile normalized these measurements and performed PCA to account for unmeasured confounders. For the eQTL analyses, we again found the most QTL signal when 13 PCs were regressed out. The eQTL analyses were performed by testing for association between mean expression levels and SNPs or indels with a minimum allele frequency greater than 10%, using the following model:
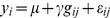
(4)where *y*_i_ is defined as in model (2) and g_ij_ corresponds to the genotype of individual *i* at variant *j*. In this model, the coefficient *γ* indicates the strength of association between the mean steady-state expression level of the gene and genotypes at variant *j*. FDR calculations were performed as described above.

To assess whether the enrichment of significant mRNA decay effects among eQTL SNPs could occur by random chance, we performed a permutation based significance test. Specifically, we evaluated the effect of genotype on mRNA decay variation using the most significant cis-eQTL SNP for all genes in our dataset (regardless of the genome-wide significance of the SNP). Then, we randomly chose 1,257 SNPs from this full set (representing the number of genome wide significant eQTLs identified) and calculated the number that showed significant association with mRNA decay variation among this set. We also ensured that the distribution of gene expression levels associated with the randomly sampled SNPs matched the distribution of expression levels for genes with significant eQTLs. By repeating this 1,000 times, we were able to arrive at a permutation-based expectation for the enrichment of significant mRNA decay effects among eQTL SNPs.

In order to look at overlaps between the set of identified rdQTLs and previously identified dsQTLs, we focused on the set of 66 YRI LCLs that were used in both studies. Using mean gene expression measures from this study, we re-mapped eQTLs as described above in this set of 66 LCLs and identified 1,147 steady-state eQTLs (15% FDR). Using these 1,147 eQTL SNPs, we tested for association between each SNP and DNaseI sensitivity as described previously [32] and between each SNP and RNA decay rates (as described above). To obtain an estimate of the total proportion of eQTLs we might be able to account for by either RNA decay variation or variation in DNaseI sensitivity, we assessed, for each SNP, the evidence for association with either data type. We then chose the minimum p-value for the association with decay rates or DNaseI sensitivity and compared the resulting distribution to the following analytical transformation:
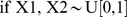


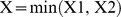


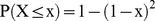
We then applied the Storey *et al.* qvalue approach to account for incomplete power [48] to this transformed distribution of p-values.

### Data submission

All raw data and tables of all rdQTLs are available under GEO accession number GSE37451.

## Supporting Information

Figure S1 Distributions of the amount of total RNA extracted across individuals from increasing cell quantities over time. In order to account for the decrease in total RNA due to the Act-D treatment, we increased the amount of cells from which we extracted RNA over time (x-axis). This allowed us to obtain similar amounts of total RNA (y-axis) for each time point, with no significant differences in median levels of total RNA (across individuals) for each time point.
(TIF)

Figure S2 Examples of gene-specific mRNA decay data from pilot experiments across 5 cell lines. In every plot, time course (x-axis) estimates of normalized (un-transformed) gene expression levels (y-axis) from each of the five cell lines are plotted. The top panels show examples of genes whose transcripts decay at a rate similar to the mean decay rate in the cell lines. The observed pattern of no apparent decay is a result of our normalization approach. To visualize decay, we standardize (described in the main paper) the normalized expression values by the number of cells from which RNA was extracted at each time point. The bottom panels show two examples of genes decaying faster (left) or slower (right) than average. It is evident that the later time points (8 and 12 hours) do not provide significant additional information to the decay fit when compared to earlier time points.
(TIF)

Figure S3 Boxplots of distributions of pairwise correlations. Pearson correlations (y-axis) are plotted for (from left to right on the x-axis): biological replicates (from the pilot experiment data), data from different time points of the same cell line (from full dataset), data from different cell lines for the same time points (from full dataset), and data from different cell lines across time points (from full dataset).
(TIF)

Figure S4 Influence of gene length on decay rates after accounting for 3′UTR length. Distributions of non-3′UTR region gene lengths (y-axis) for slow decaying genes (blue) and fast decaying genes (red).
(TIF)

Figure S5 Significant difference between expression levels of slow decaying genes and fast decaying genes. Genome-wide scatterplot of median decay rates (x-axis) versus median steady-state expression levels (y-axis). Colors of the regions indicate the density of points (higher density in darker colors). The yellow circle indicates *BTG1*, an example of a gene with a high decay rate and high expression level.
(TIF)

Figure S6 Distribution of PolII ChIP-seq tags in gene body regions. Increase in the density (y-axis) of positive Pearson correlations (x-axis) for genes with discordant (purple) compared to concordant (orange) relationship between mRNA decay rates and gene expression levels.
(TIF)

Figure S7Estimates of the proportion of most significant eQTL SNPs that are significantly associated with decay rates. All analyses are done using the R package ‘qvalue’ as described in Storey and Tibshirani 2003. A. Estimated fraction of test statistics (π_0_) that are generated under the null hypothesis (no association with decay), as a function of the tuning parameter λ (solid line). The 95% bootstrap confidence band is also shown (dashed lines). The vertical dashed line corresponds to λ for which the bootstrap mean square error for the estimate of 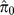 is the smallest. B. Distribution of the p-values for tests of association with decay rates and the distribution that would be expected if all test statistics were generated under the null hypothesis (no association with decay) π_0_ = 1 (dashed red line), and the fraction (solid red line) of null tests estimated to be present from the observed sample.(TIF)

Figure S8 Evidence for association with decay and expression for SNPs in functionally annotated regions. A. The QQ-plots of expected versus observed quantiles of the –log10(p-values) for association with decay for SNPs located in coding exons (green), 5′UTRs (dark red), 3′UTRs (red), promoter regions (5 kb upstream of TSS; in orange), and all other intergenic and intronic SNPs (black). B. The QQ-plots of expected versus observed quantiles of the –log10(p-values) for association with expression for all 3′UTR SNPs (red) and in two known 3′UTR functional annotations – predicted miRNA binding sites (dark blue) and AU-rich element pentamers (light blue).
(TIF)

Figure S9 Evaluating factors causing bias in the estimation of the proportion of eQTLs also classified as rdQTLs. A. Boxplots of the distribution of the total number of SNPs in all cis-candidate windows for genes with only eQTLs (left) and genes with eQTLs that are also rdQTLs (right). B. Boxplots of the distribution of minor allele frequencies for SNPs identified as only eQTLs (right) or eQTLs that are also rdQTLs (right). C. Boxplots of the distribution of the number of minor allele homozygotes for SNPs identified as only eQTLs (right) or eQTLs that are also rdQTLs (right).
(TIF)

Figure S10 Numbers of eQTLs that are also classified as rdQTLs (right), dsQTLs (left), or both (middle).
(TIF)

Figure S11Estimates of the proportion of most significant eQTL SNPs that are associated with either decay rates or DNaseI sensitivity. All analyses are done using the R package ‘qvalue’ as described in Storey and Tibshirani 2003. A. Estimated fraction of test statistics (π_0_) that are generated under the null hypothesis (no association with either decay or DNaseI sensitivity), as a function of the tuning parameter λ (solid line). The 95% bootstrap confidence band is also shown (dashed lines). The vertical dashed line corresponds to λ for which the bootstrap mean square error for the estimate of 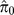 is the smallest. B. Distribution of the transformed minimum p-values for tests of association with either decay rates or DNaseI sensitivity and the distribution that would be expected if all test statistics were generated under the null hypothesis (no association with decay or DNaseI sensitivity) π_0_ = 1 (dashed red line), and the fraction (solid red line) of null tests estimated to be present from the observed sample.(TIF)

Methods S1 Supplementary materials and methods for analyses presented in the main text.
(DOC)

Table S1 Summary of data for expression timecourse and decay rate calculations.
(TXT)

Table S2 Gene Ontology categories for genes decaying slower than average (FDR<0.1%)
(XLSX)

Table S3 Gene Ontology categories for genes decaying faster than average (FDR<0.1%).
(XLSX)

Table S4 Gene Ontology categories for the 47 genes in the top 10% of both decay and gene expression distributions (FDR<1%).
(XLSX)

Table S5 Gene Ontology categories for genes with a concordant relationship between decay and gene expression across individuals (FDR<0.1%).
(XLSX)

Table S6 Gene Ontology categories for genes with a discordant relationship between decay and gene expression across individuals (FDR<0.1%).
(XLSX)

Table S7 Summary of data for PolII ChIP-seq reads.
(XLSX)

Table S8 Information on rdQTLs identified in this study.
(TXT)
