# Supplementary figures and images for: The Contribution of RNA Decay Quantitative Trait Loci to Inter-Individual Variation in Steady-State Gene Expression Levels
Source: PLoS Genet. 2012 Oct 11;8(10):e1003000. doi: 10.1371/journal.pgen.1003000 (PMC3469421; doi:10.1371/journal.pgen.1003000)

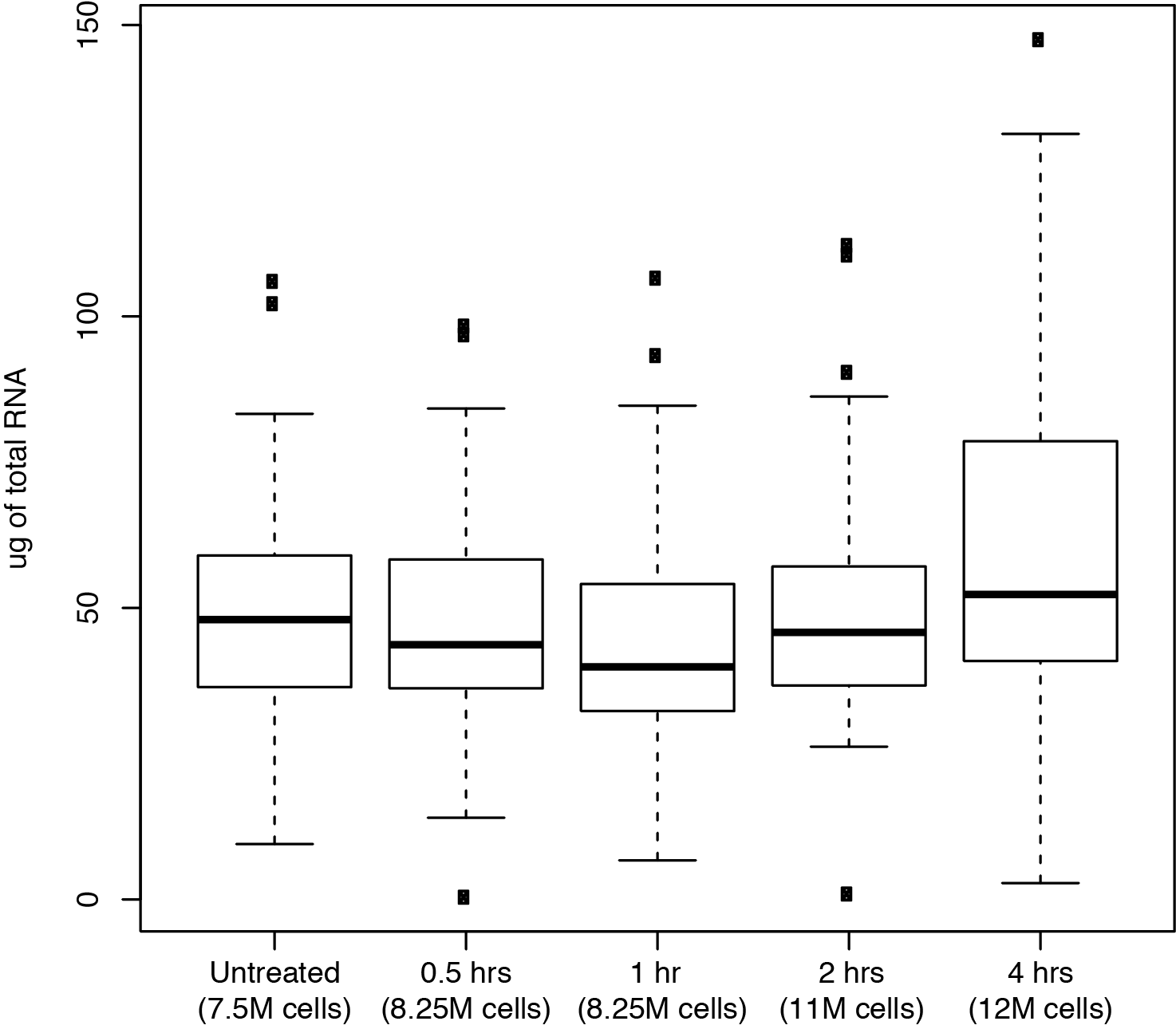

Supplement: Figure S1 — Distributions of the amount of total RNA extracted across individuals from increasing cell quantities over time. In order to account for the decrease in total RNA due to the Act-D treatment, we increased the amount of cells from which we extracted RNA over time (x-axis). This allowed us to obtain similar amounts of total RNA (y-axis) for each time point, with no significant differences in median levels of total RNA (across individuals) for each time point. (TIF) [file pgen.1003000.s001.tif]

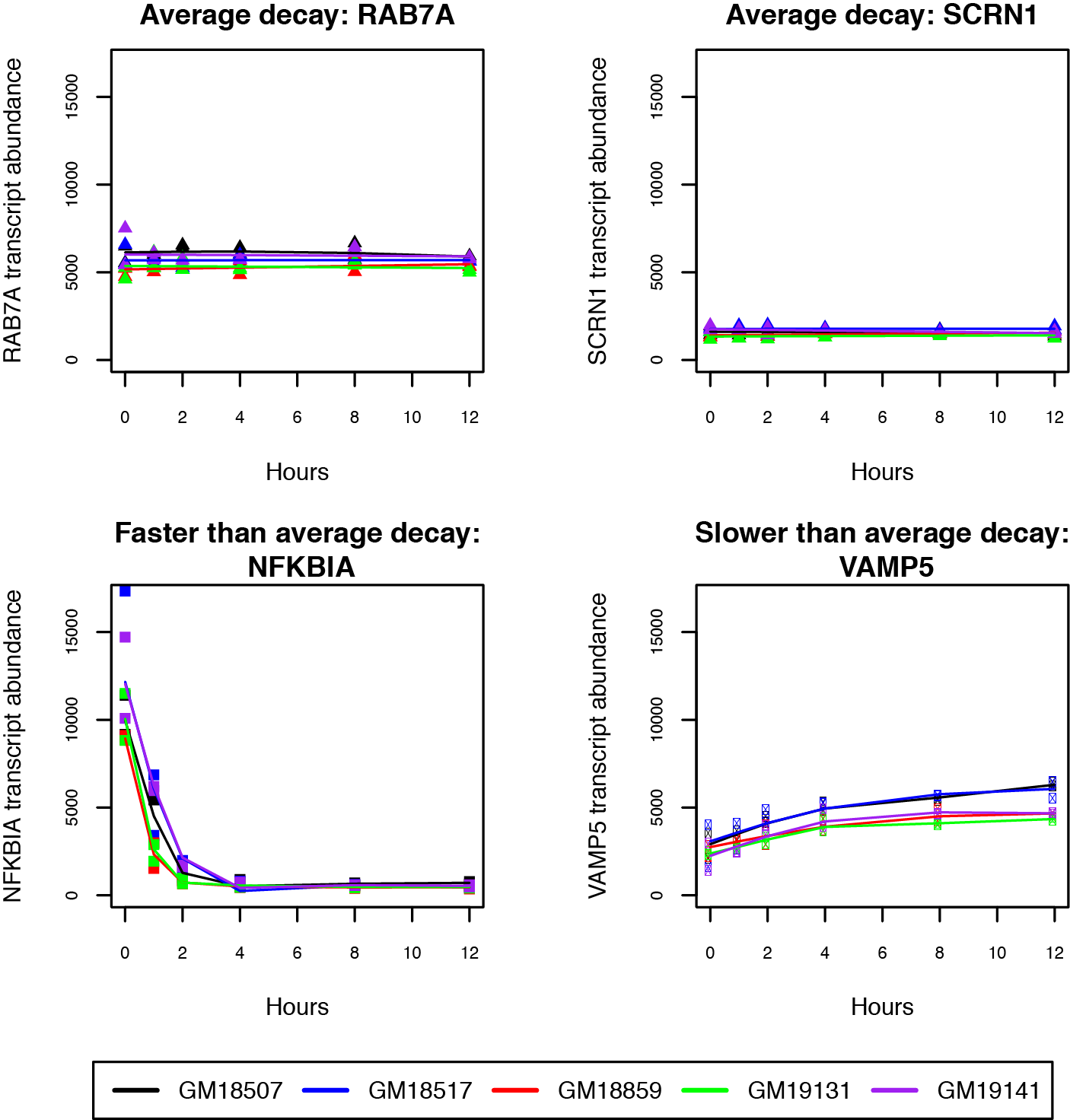

Supplement: Figure S2 — Examples of gene-specific mRNA decay data from pilot experiments across 5 cell lines. In every plot, time course (x-axis) estimates of normalized (un-transformed) gene expression levels (y-axis) from each of the five cell lines are plotted. The top panels show examples of genes whose transcripts decay at a rate similar to the mean decay rate in the cell lines. The observed pattern of no apparent decay is a result of our normalization approach. To visualize decay, we standardize (described in the main paper) the normalized expression values by the number of cells from which RNA was extracted at each time point. The bottom panels show two examples of genes decaying faster (left) or slower (right) than average. It is evident that the later time points (8 and 12 hours) do not provide significant additional information to the decay fit when compared to earlier time points. (TIF) [file pgen.1003000.s002.tif]

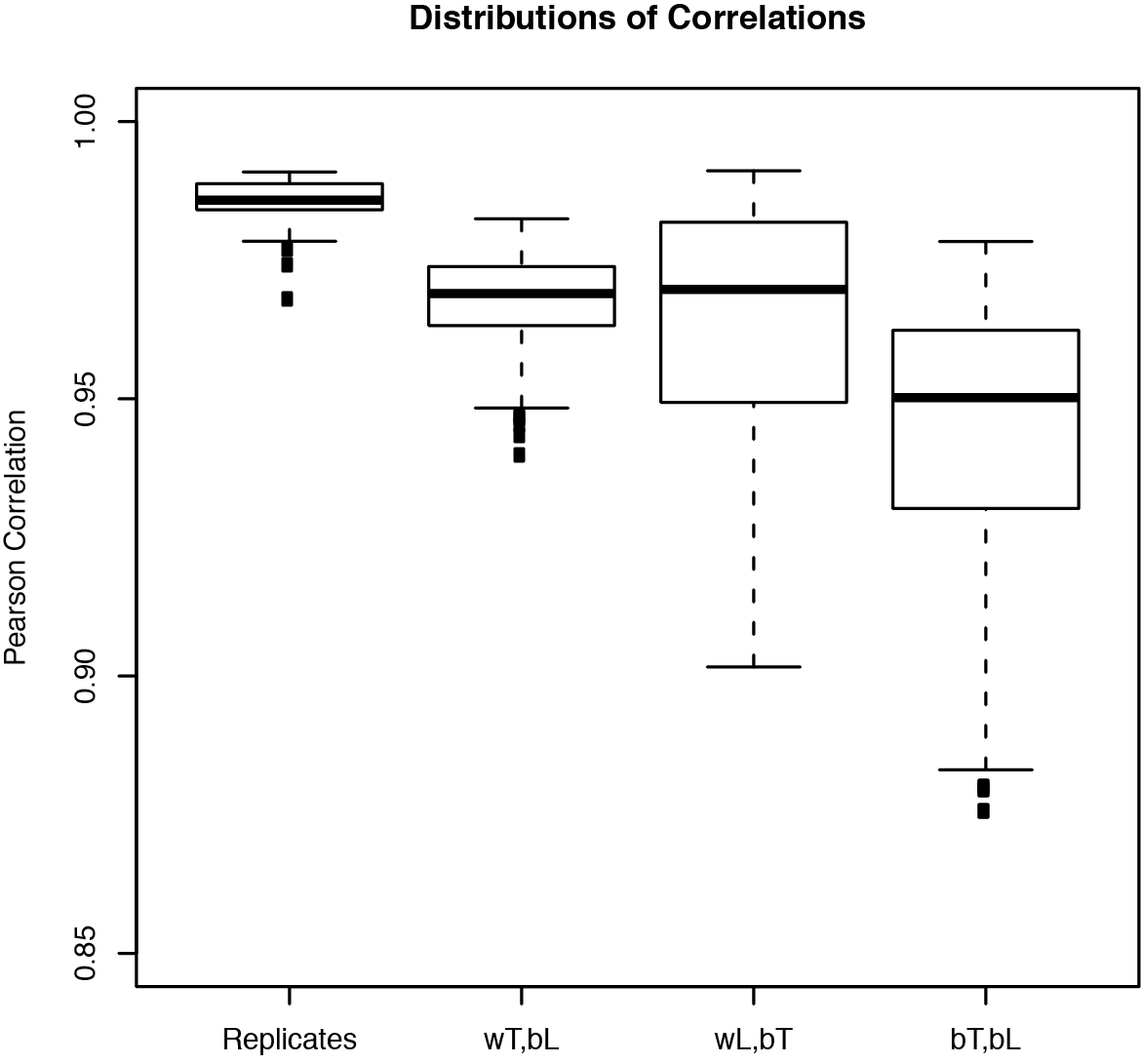

Supplement: Figure S3 — Boxplots of distributions of pairwise correlations. Pearson correlations (y-axis) are plotted for (from left to right on the x-axis): biological replicates (from the pilot experiment data), data from different time points of the same cell line (from full dataset), data from different cell lines for the same time points (from full dataset), and data from different cell lines across time points (from full dataset). (TIF) [file pgen.1003000.s003.tif]

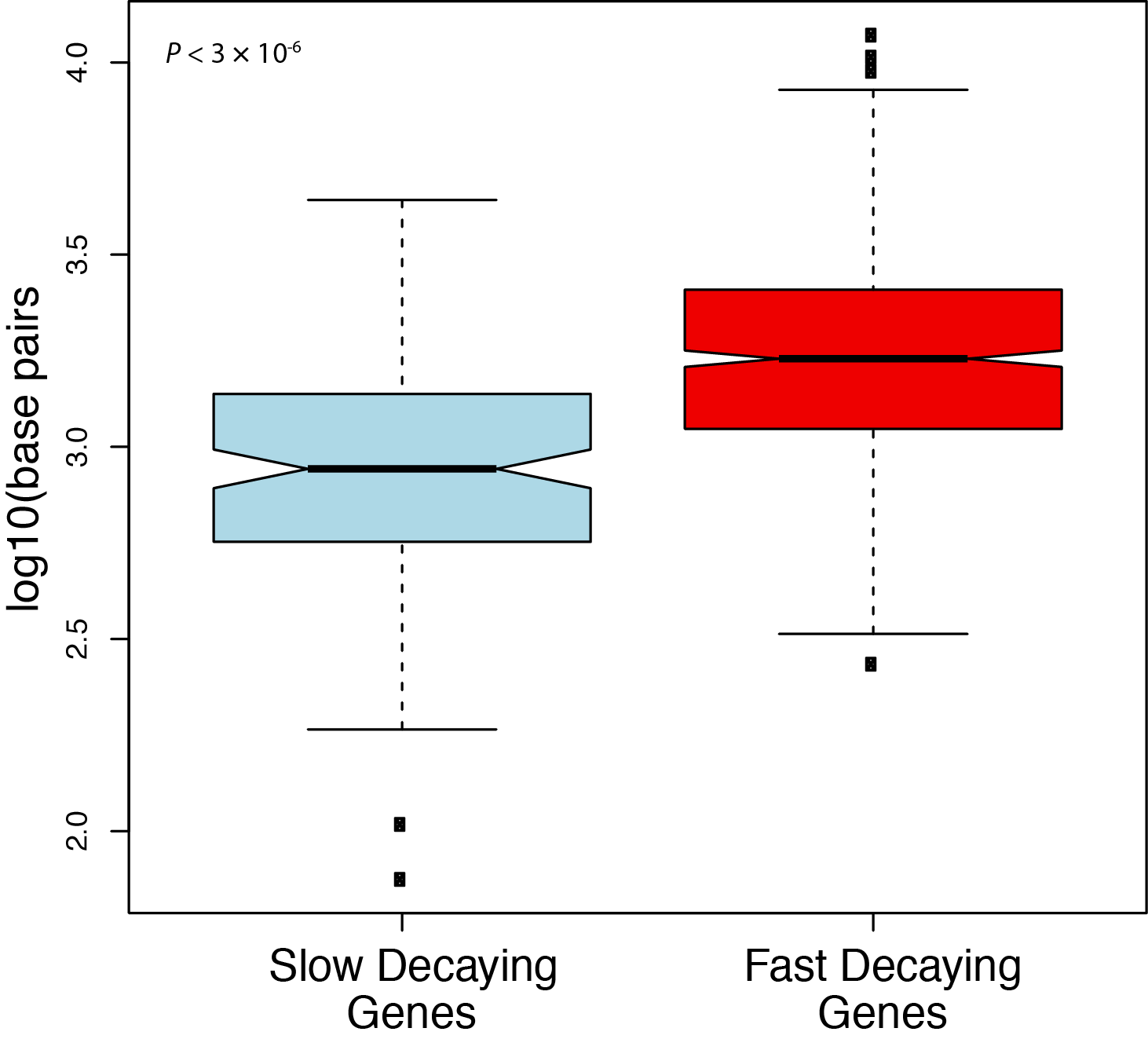

Supplement: Figure S4 — Influence of gene length on decay rates after accounting for 3′UTR length. Distributions of non-3′UTR region gene lengths (y-axis) for slow decaying genes (blue) and fast decaying genes (red). (TIF) [file pgen.1003000.s004.tif]

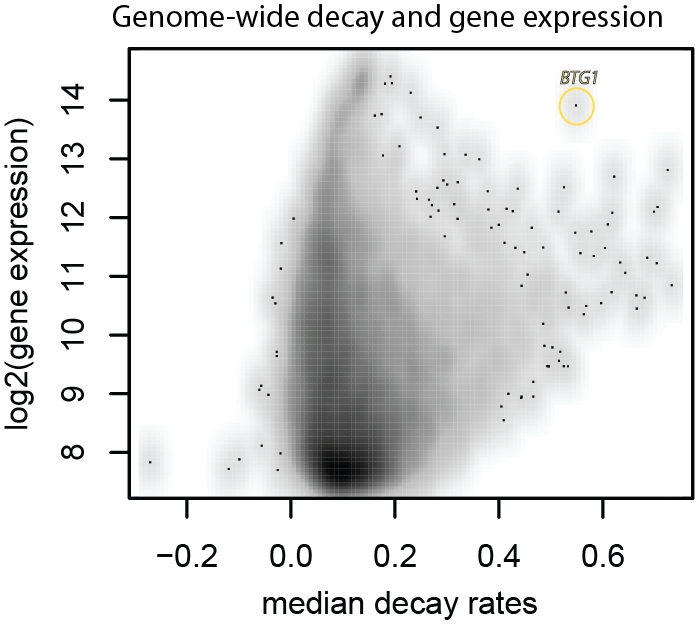

Supplement: Figure S5 — Significant difference between expression levels of slow decaying genes and fast decaying genes. Genome-wide scatterplot of median decay rates (x-axis) versus median steady-state expression levels (y-axis). Colors of the regions indicate the density of points (higher density in darker colors). The yellow circle indicates BTG1, an example of a gene with a high decay rate and high expression level. (TIF) [file pgen.1003000.s005.tif]

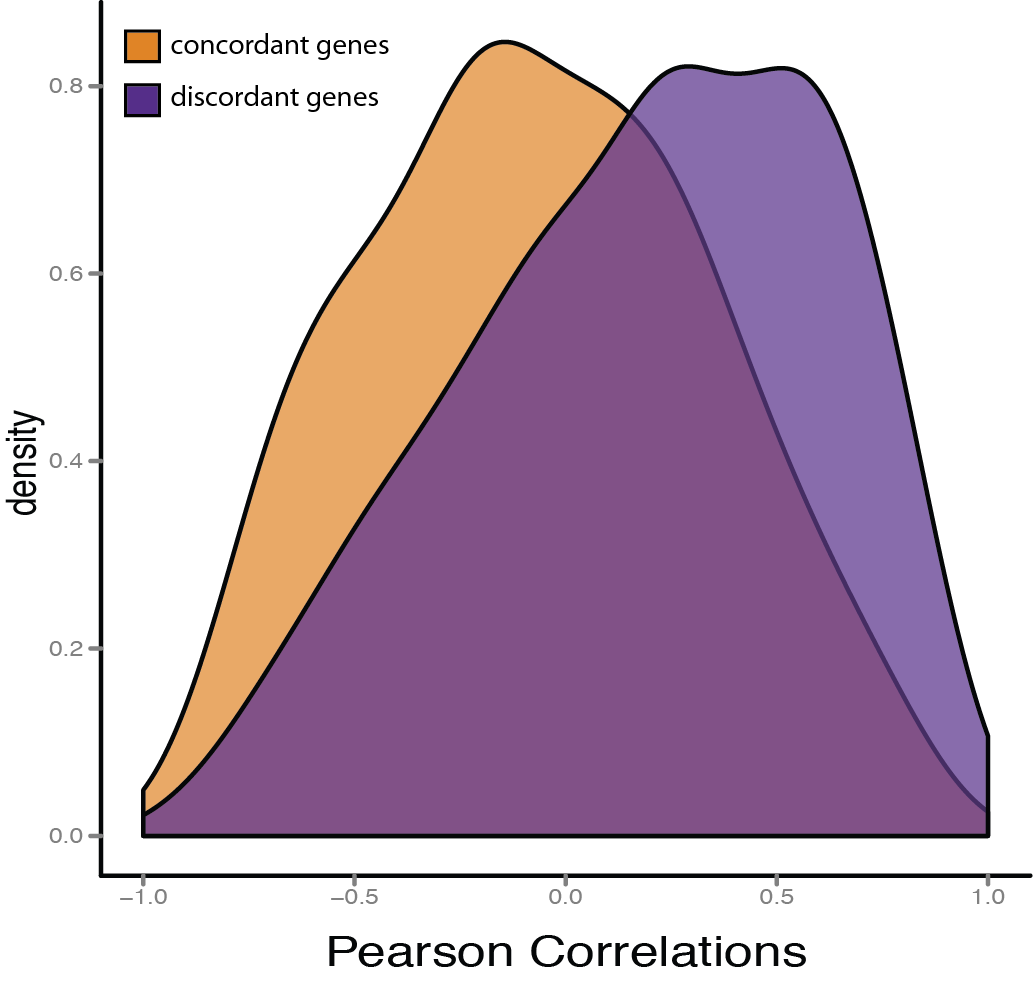

Supplement: Figure S6 — Distribution of PolII ChIP-seq tags in gene body regions. Increase in the density (y-axis) of positive Pearson correlations (x-axis) for genes with discordant (purple) compared to concordant (orange) relationship between mRNA decay rates and gene expression levels. (TIF) [file pgen.1003000.s006.tif]

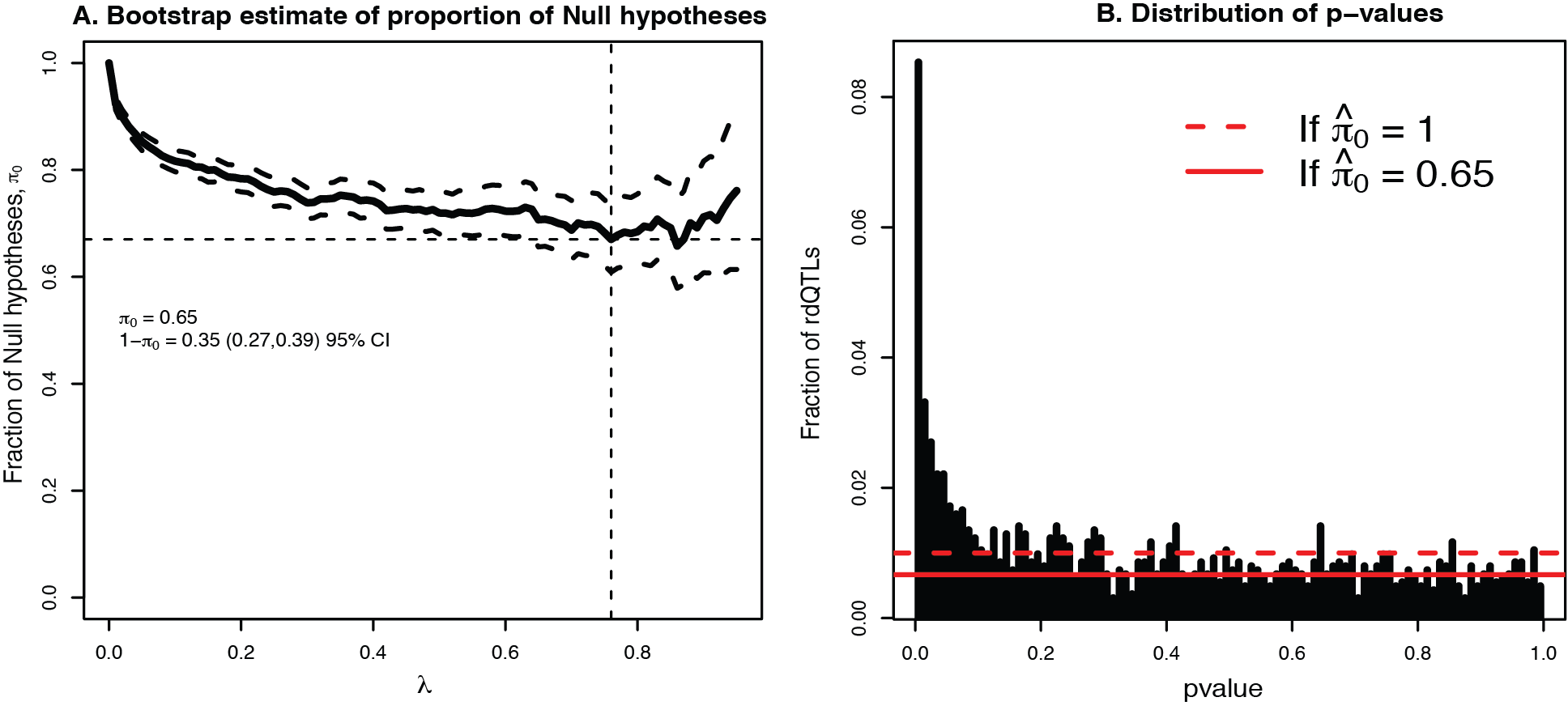

Supplement: Figure S7 — Estimates of the proportion of most significant eQTL SNPs that are significantly associated with decay rates. All analyses are done using the R package ‘qvalue’ as described in Storey and Tibshirani 2003. A. Estimated fraction of test statistics (π0) that are generated under the null hypothesis (no association with decay), as a function of the tuning parameter λ (solid line). The 95% bootstrap confidence band is also shown (dashed lines). The vertical dashed line corresponds to λ for which the bootstrap mean square error for the estimate of is the smallest. B. Distribution of the p-values for tests of association with decay rates and the distribution that would be expected if all test statistics were generated under the null hypothesis (no association with decay) π0 = 1 (dashed red line), and the fraction (solid red line) of null tests estimated to be present from the observed sample. (TIF) [file pgen.1003000.s007.tif]

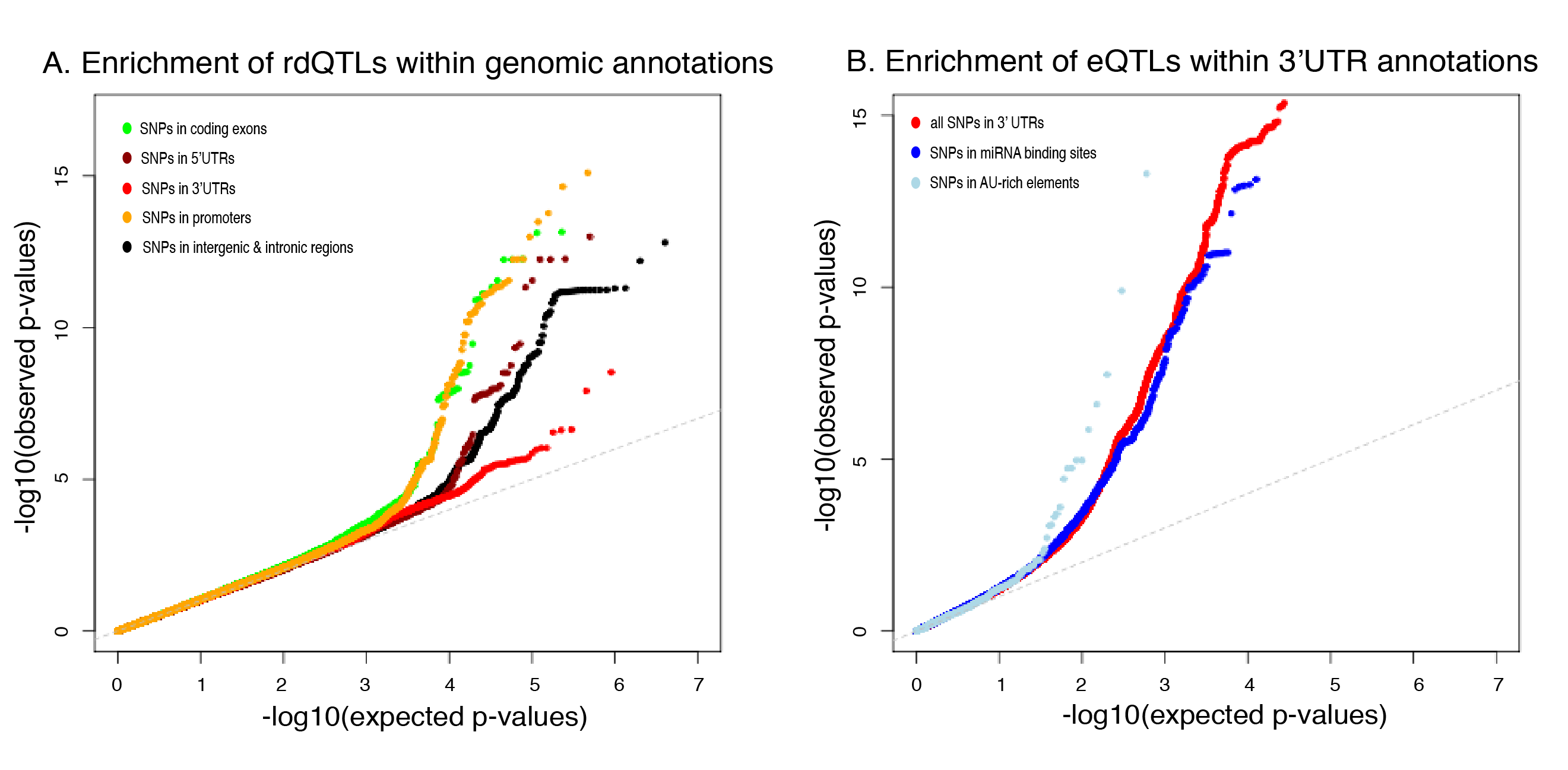

Supplement: Figure S8 — Evidence for association with decay and expression for SNPs in functionally annotated regions. A. The QQ-plots of expected versus observed quantiles of the –log10(p-values) for association with decay for SNPs located in coding exons (green), 5′UTRs (dark red), 3′UTRs (red), promoter regions (5 kb upstream of TSS; in orange), and all other intergenic and intronic SNPs (black). B. The QQ-plots of expected versus observed quantiles of the –log10(p-values) for association with expression for all 3′UTR SNPs (red) and in two known 3′UTR functional annotations – predicted miRNA binding sites (dark blue) and AU-rich element pentamers (light blue). (TIF) [file pgen.1003000.s008.tif]

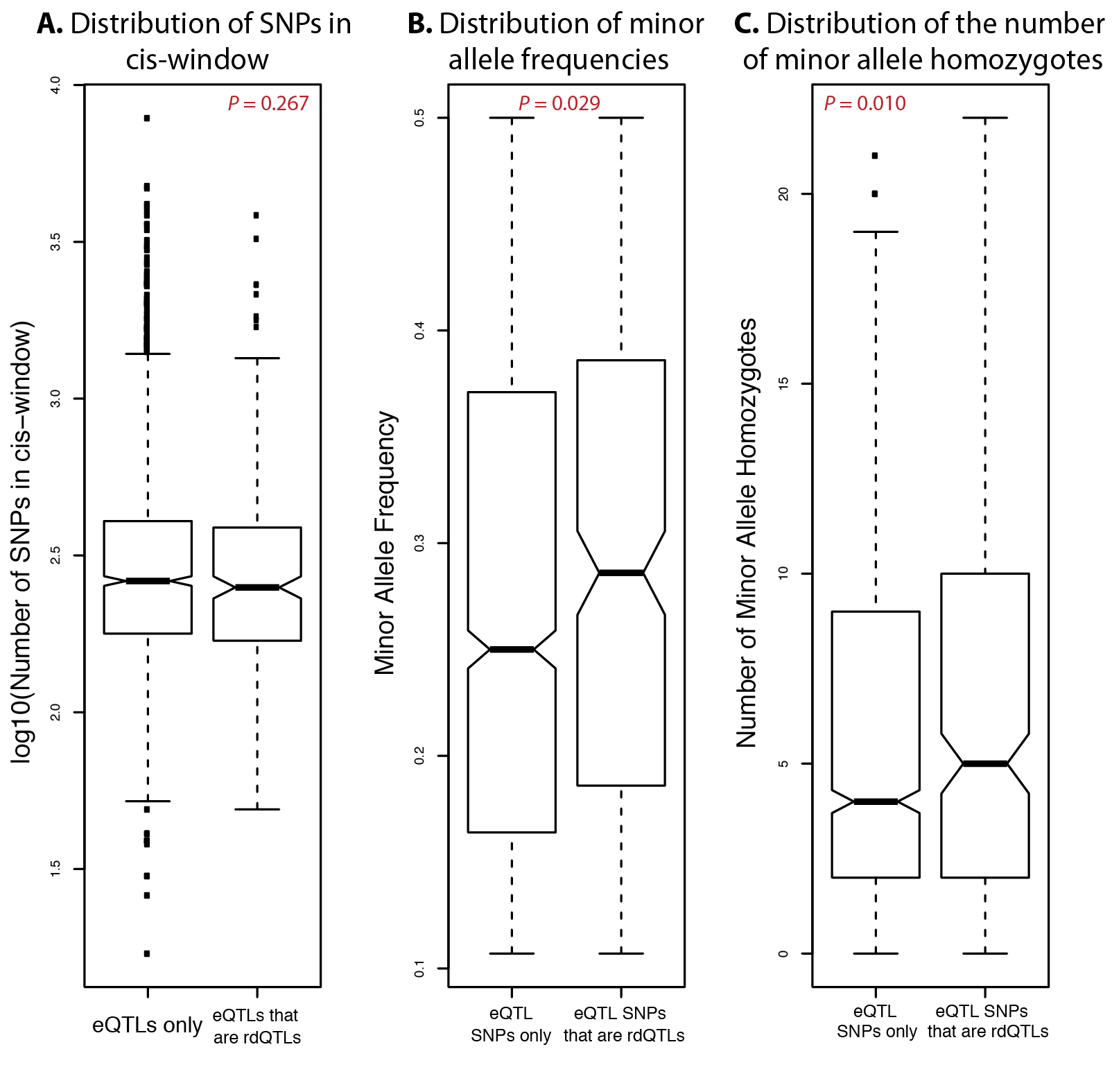

Supplement: Figure S9 — Evaluating factors causing bias in the estimation of the proportion of eQTLs also classified as rdQTLs. A. Boxplots of the distribution of the total number of SNPs in all cis-candidate windows for genes with only eQTLs (left) and genes with eQTLs that are also rdQTLs (right). B. Boxplots of the distribution of minor allele frequencies for SNPs identified as only eQTLs (right) or eQTLs that are also rdQTLs (right). C. Boxplots of the distribution of the number of minor allele homozygotes for SNPs identified as only eQTLs (right) or eQTLs that are also rdQTLs (right). (TIF) [file pgen.1003000.s009.tif]

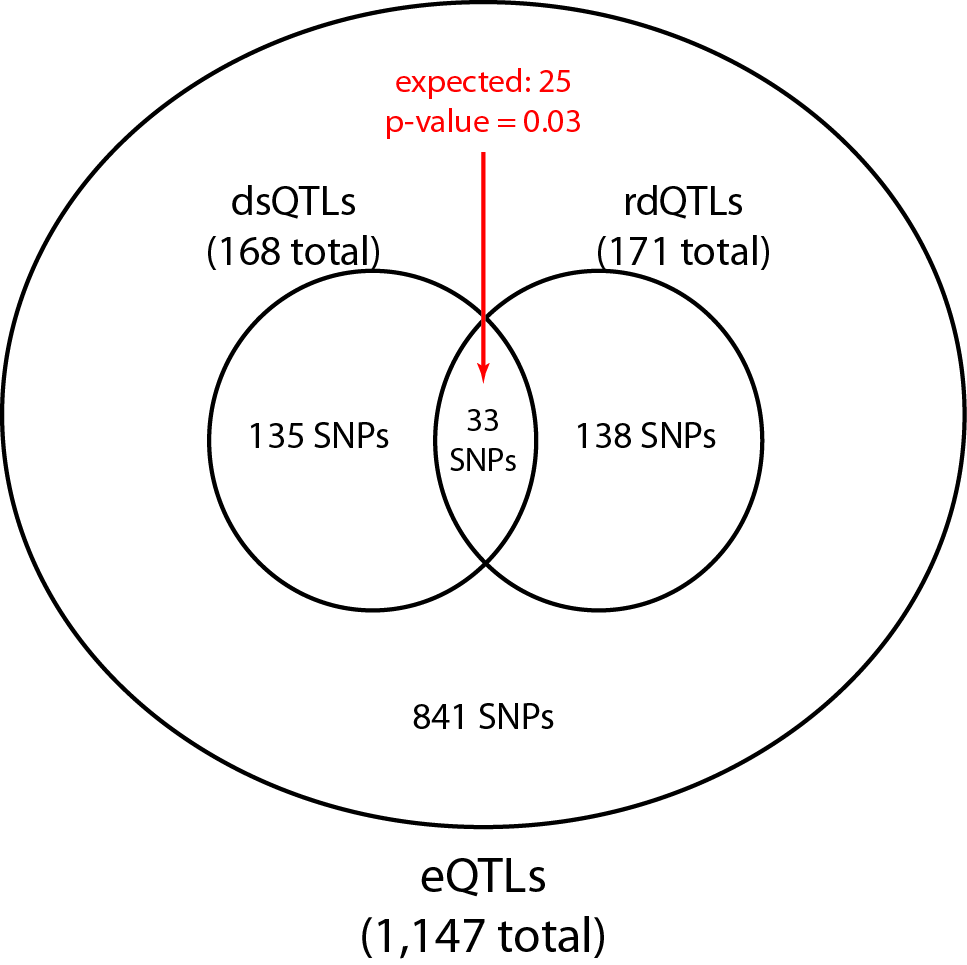

Supplement: Figure S10 — Numbers of eQTLs that are also classified as rdQTLs (right), dsQTLs (left), or both (middle). (TIF) [file pgen.1003000.s010.tif]

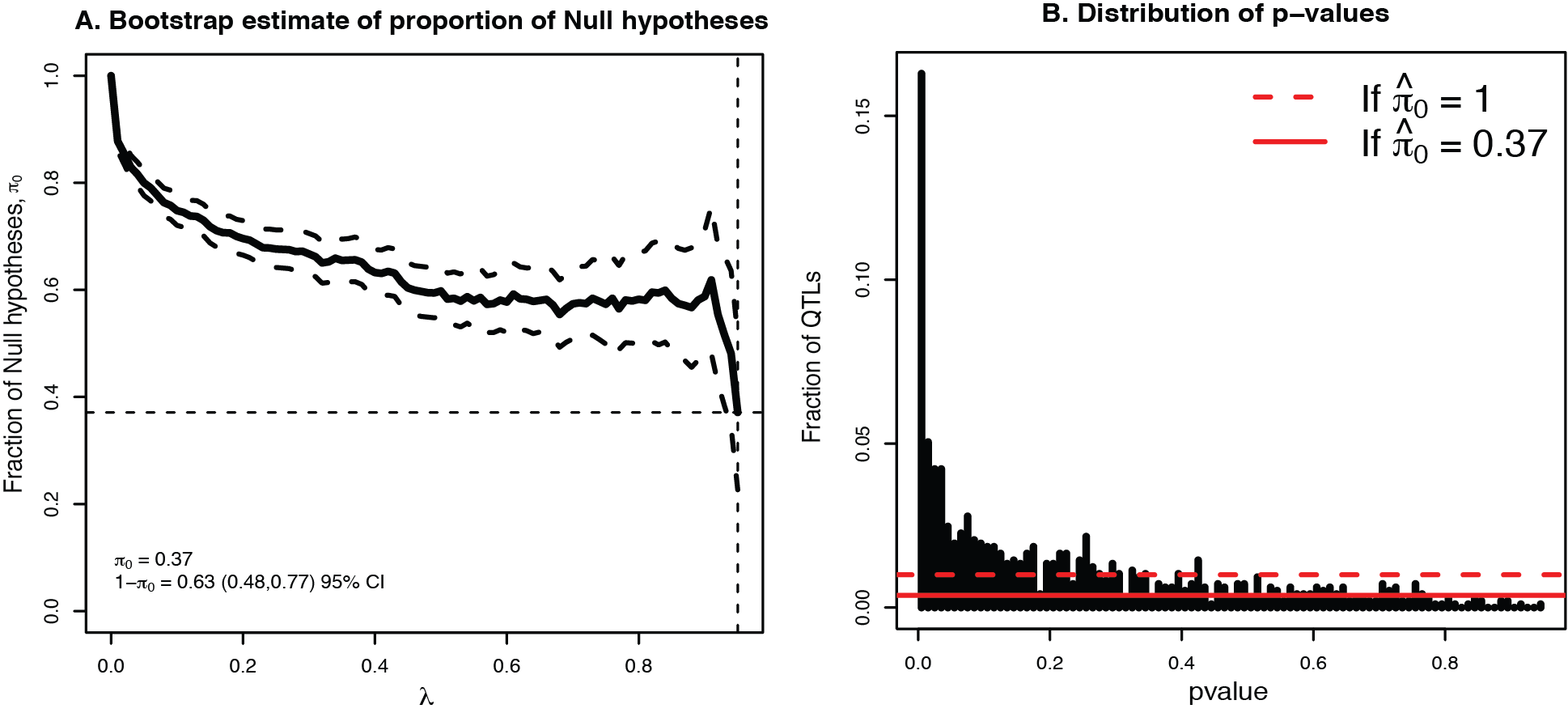

Supplement: Figure S11 — Estimates of the proportion of most significant eQTL SNPs that are associated with either decay rates or DNaseI sensitivity. All analyses are done using the R package ‘qvalue’ as described in Storey and Tibshirani 2003. A. Estimated fraction of test statistics (π0) that are generated under the null hypothesis (no association with either decay or DNaseI sensitivity), as a function of the tuning parameter λ (solid line). The 95% bootstrap confidence band is also shown (dashed lines). The vertical dashed line corresponds to λ for which the bootstrap mean square error for the estimate of is the smallest. B. Distribution of the transformed minimum p-values for tests of association with either decay rates or DNaseI sensitivity and the distribution that would be expected if all test statistics were generated under the null hypothesis (no association with decay or DNaseI sensitivity) π0 = 1 (dashed red line), and the fraction (solid red line) of null tests estimated to be present from the observed sample. (TIF) [file pgen.1003000.s011.tif]
