# Supplementary material for: The Contribution of RNA Decay Quantitative Trait Loci to Inter-Individual Variation in Steady-State Gene Expression Levels
Source: PLoS Genet. 2012 Oct 11;8(10):e1003000. doi: 10.1371/journal.pgen.1003000 (PMC3469421; doi:10.1371/journal.pgen.1003000)
Supplement: Methods S1 — Supplementary materials and methods for analyses presented in the main text. (DOC) [file pgen.1003000.s012.doc]

**Supplementary Methods for**

**The contribution of RNA decay quantitative trait loci to inter-individual variation in steady-state gene expression levels**

Athma A. Pai^1^, Carolyn E. Cain^1^, Orna Mizrahi-Man^1^, Sherryl De Leon^2^, Noah Lewellen^2^, Jean-Baptiste Veyrieras^1,3^, Jacob F. Degner^1,4^, Daniel J. Gaffney^1,2^, Joseph K. Pickrell^1^, Matthew Stephens^1,5^, Jonathan K. Pritchard^1,2^, Yoav Gilad^1^

^1^ Department of Human Genetics, University of Chicago, Chicago, IL 60637

^2^ Howard Hughes Medical Institute, University of Chicago, Chicago, IL 60637

^3^ BioMiningLabs, Lyon, France, 69001

^4^ Committee on Genetics, Genomics, and Systems Biology, University of Chicago, Chicago, IL 60637

^5^ Department of Statistics, University of Chicago, Chicago, IL 60637

Pilot experiments to calibrate treatments and time points

Previous studies suggested that the mean half-life of a subset of human transcripts can be as long as 8-10 hours [1,2]. In light of this, we conducted an initial pilot study to choose appropriate time points. In this pilot, we treated 5 cell lines with ActD in two treatment replicates and extracted RNA from the following time points: 0 hours (prior to treatment), 1 hour, 2 hours, 4 hours, 8 hours, and 12 hours following treatment (resulting in a total of 60 samples). We estimated gene expression levels in all samples by hybridizing RNA to Illumina HT-12 arrays, normalized the data across all arrays using standard normalization methods, and estimated decay rates by fitting a first order exponential decay model across time points (see Methods for full details). We estimated relative gene-specific RNA decay rates using different combinations of subsets of the data (from subsets of the time points) to determine the most informative design for the larger study. Using this approach, we found that none of the early time points provided redundant information, prompting us to add an earlier time point – 0.5 hours – for the larger study (Figure S1). In contrast, inclusion of data from the later time points (8 and 12 hours) resulted in a poorer fit of the decay model, prompting us to exclude these time points from the larger study. Finally, as we performed the pilot experiments in two replicates from each of the cell lines, we were able to use the estimated gene-specific RNA decay rates to assess variation associated with the treatment itself. We found that variation due to the treatment is considerably lower than variation across time points or across cell lines (Figure S3). We thus proceeded with only one replicate per cell line in the larger study.

Parsing miRNA binding databases

In order to create a full set of microRNA (miRNA) binding site predictions, we downloaded (in August 2011) and pulled the miRNA binding predictions from three databases: microRNA.org [http://www.microrna.org/], PicTar [http://pictar.mdc-berlin.de/], and targetScan [<http://www.targetscan.org/vert_50/>]. All databases from which we downloaded miRNA binding site predictions relate microRNAs to RefSeq or Genbank [3] identifiers, while probes on our microarray were mapped to Ensembl gene identifiers [4]. Thus, we converted the RefSeq and Genbank transcript identifiers to Ensembl Release 63 identifiers using Ensembl BioMart [5]. We also converted the 16,823 Ensembl Release 54 gene identifiers from our microarray gene set to Ensembl Release 63 identifiers, resulting in a total set of 16,239 genes with Ensembl Release 63 counterparts. Finally, we filtered out all Genbank and RefSeq transcripts that mapped to more than one location in the genome using flat files downloaded from the UCSC genome browser database [6].

*Obtaining and processing predictions from microRNA.org*: Human target site prediction files from the Aug. 2010 database release were downloaded from the microRNA.org database (<http://www.microrna.org/>) [7]. We applied three filtering steps to obtain a final file with an Ensembl gene identifier, miRNA, and genomic coordinates of a predicted binding site: (1) We extracted the genomic coordinates of the binding sites from microRNA.org and trimmed them to the region that aligns between the miRNA and gene. Because the presence of an “A” at the position that aligns with the first nucleotide of the miRNA has been predicted to be of functional importance [8], we extended the binding site to include the nucleotide that would bind the first miRNA nucleotide in cases where (a) the gene had an “A” at this position and (b) the region aligned between the gene and miRNA included the miRNA’s second nucleotide. (2) We converted the RefSeq or GenBank identifiers used in microRNA.org to Ensembl 54 identifiers (as described above), resulting in the retention of 8,051,359/16,228,619 total entries. (3) We used liftOver [9] to convert the coordinates in microRNA.org, which related to human genome assembly hg19 to hg18 coordinates (because the probes on the array we used were designed based on hg18 coordinates). Since not all coordinates could be converted, this resulted in (using the categories reported by the database): 581,243 “Good mirSVR score, Conserved miRNA”, 1,718,280 “Good mirSVR score, Non-conserved miRNA”, 1,298,2,11 “Non-good mirSVR score, Conserved miRNA”, and 4,450,030 “Non-good mirSVR score, Non-conserved miRNA” binding site predictions.

*Obtaining and processing PicTar predictions*: Track files containing miRNA binding site predictions generated by the PicTar algorithm [10,11] were downloaded from the hg17 archive of the UCSC genome browser database [6]. Two files with different prediction stringencies were downloaded: (a) conservation among four vertebrates: human, mouse, rat, and dog, and (b) conservation among five vertebrates: human, mouse, rat, dog, and chicken. We applied the following filtering steps to obtain a final list: (1) We converted the RefSeq identifiers used by PicTar to Ensembl 54 identifiers (as described above). We also changed, wherever necessary, the PicTar suffixes to conform with the miR names in miRbase [12]. (2) We used liftOver [9] to convert the hg17 coordinates in the PicTar entries to hg18 coordinates. This resulted in a final set of 131,542 and 26,585 binding site predictions for the four and five species sets, respectively.

*Obtaining and processing targetScan predictions*: Prediction files were downloaded from the targetScan website (<http://www.targetscan.org/vert_50/>) [8,13,14], and we extracted the subset of predictions that pertain to humans from all files. We applied the following filtering steps to obtain a final list: (1) Because targetScan predictions are provided as positions on the gene’s 3’ UTR sequence, we converted these positions into genomic coordinates by mapping the UTR sequences downloaded from targetScan onto hg19. After parsing out entries with little to no overlap with known 3’UTR sequences from RefSeq definitions and overlaps with multiple known RefSeq 3’UTRs, we obtained a final set of 17,481 gene 3’ UTR definitions corresponding to targetScan 3’ UTRs. (2) To match the targetScan predictions with data from other databases, we (a) converted the gene symbols from targetScan files to Ensembl Release 54 identifiers, (b) converted the coordinates relative to the targetScan 3’UTR sequences to genomic coordinates (as described in the previous step), and (c) converted the miRNA family definitions to individual human miRNA identifiers, which was necessary because targetScan predictions are given per miRNA family. For this last step, we used the “miR families” file downloaded from the targetScan website to create multiple prediction records from each predicted binding site – one for each member of the miR family. (3) We used liftOver [9] to convert the hg19 coordinates to hg18. These resulted in a final set of sites comprising of (using the categories reported by the database): 43,380 predicted conserved target of conserved miRNA families, 359,946 conserved and non-conserved sites corresponding to conserved miRNA families, and 827,068 conserved and non-conserved sites corresponding to non-conserved miRNA families.

We merged overlapping predictions from the three databases, conditional on pertaining to the same gene and miRNA. When a site was predicted by more than one database we considered the union of predicted regions. Overall, we obtained 8,132,475 individual miRNA binding sites in 3’ UTRs of 6,898,569 non-overlapping loci (once we merged overlapping predictions).

Analysis of *trans*-effects on variation in mRNA decay rates

Mechanisms of mRNA decay often involve *trans*-acting factors, such as RNA-binding proteins, which can significantly influence mRNA decay rates [15]. We hypothesized that genetic variation associated with either the expression levels or changes to the coding sequences of genes encoding these decay-related proteins could significantly affect mRNA decay rates of many target genes (namely, these sites are likely to be trans rdQTLs). To test this hypothesis, we conducted *trans*-QTL mapping by limiting our analyses to the biologically likely candidate regions. We did this by only testing candidate SNPs within or around genes encoding known mammalian RNA-binding proteins or decay factors (2,216 Ensembl genes, of which 1,688 are autosomal genes and expressed within our dataset). Using the model for QTL association described in the Methods, we tested for evidence of *trans*-rdQTLs by: (1) testing for association between the most significant eQTL SNPs for each of the 1,688 candidate genes and all 16,823 gene-specific decay rates, and (2) testing for association between all exonic SNPs within each of the 1,688 candidate genes and all 16,823 gene-specific decay rates. In both cases, we were unable to identify any significant QTLs at an FDR of 15%, most likely because our experiment is underpowered to detect *trans* effects. The most significant *trans-*rdQTL (rs7965292; an eQTL for the DDX55 gene) was associated with an FDR of 33.3%. Interestingly, the same SNP is also a *cis-*rdQTL for the DDX55 gene (see Figure 5C).

**References**

1. Yang E, van Nimwegen E, Zavolan M, Rajewsky N, Schroeder M, et al. (2003) Decay rates of human mRNAs: correlation with functional characteristics and sequence attributes. Genome Research 13: 1863–1872.

2. Raghavan A, Ogilvie RL, Reilly C, Abelson ML, Raghavan S, et al. (2002) Genome-wide analysis of mRNA decay in resting and activated primary human T lymphocytes. Nucleic Acids Res 30: 5529–5538.

3. Sayers EW, Barrett T, Benson DA, Bolton E, Bryant SH, et al. (2011) Database resources of the National Center for Biotechnology Information. Nucleic Acids Res 39: D38–51.

4. Flicek P, Amode MR, Barrell D, Beal K, Brent S, et al. (2011) Ensembl 2011. Nucleic Acids Res 39: D800–6.

5. Kinsella RJ, Kähäri A, Haider S, Zamora J, Proctor G, et al. (2011) Ensembl BioMarts: a hub for data retrieval across taxonomic space. Database (Oxford) 2011: bar030.

6. Fujita PA, Rhead B, Zweig AS, Hinrichs AS, Karolchik D, et al. (2011) The UCSC Genome Browser database: update 2011. Nucleic Acids Res 39: D876–82.

7. Betel D, Wilson M, Gabow A, Marks DS, Sander C (2008) The microRNA.org resource: targets and expression. Nucleic Acids Res 36: D149–53.

8. Lewis BP, Burge CB, Bartel DP (2005) Conserved seed pairing, often flanked by adenosines, indicates that thousands of human genes are microRNA targets. Cell 120: 15–20.

9. Hinrichs AS, Karolchik D, Baertsch R, Barber GP, Bejerano G, et al. (2006) The UCSC Genome Browser Database: update 2006. Nucleic Acids Res 34: D590–8.

10. Krek A, Grün D, Poy MN, Wolf R, Rosenberg L, et al. (2005) Combinatorial microRNA target predictions. Nat Genet 37: 495–500.

11. Lall S, Grün D, Krek A, Chen K, Wang Y-L, et al. (2006) A genome-wide map of conserved microRNA targets in C. elegans. Curr Biol 16: 460–471.

12. Kozomara A, Griffiths-Jones S (2011) miRBase: integrating microRNA annotation and deep-sequencing data. Nucleic Acids Res 39: D152–7.

13. Friedman RC, Farh KK-H, Burge CB, Bartel DP (2009) Most mammalian mRNAs are conserved targets of microRNAs. Genome Research 19: 92–105.

14. Grimson A, Farh KK-H, Johnston WK, Garrett-Engele P, Lim LP, et al. (2007) MicroRNA targeting specificity in mammals: determinants beyond seed pairing. Molecular Cell 27: 91–105.

15. Schoenberg DR, Maquat LE (2012) Regulation of cytoplasmic mRNA decay. Nat Rev Genet 13: 246–259.

16. Kasowski M, Grubert F, Heffelfinger C, Hariharan M, Asabere A, et al. (2010) Variation in Transcription Factor Binding Among Humans. Science 328: 232–235.
